# Arctiin-reinforced antioxidant microcarrier antagonizes osteoarthritis progression

**DOI:** 10.1186/s12951-022-01505-7

**Published:** 2022-06-27

**Authors:** Yang Liu, Mingzhuang Hou, Zejun Pan, Xin Tian, Zhijian Zhao, Tao Liu, Huilin Yang, Qin Shi, Xi Chen, Yijian Zhang, Fan He, Xuesong Zhu

**Affiliations:** 1 Department of Orthopaedics, The First Affiliated Hospital of Soochow University, Soochow University, No. 899 Pinghai Road, Suzhou, 215006 Jiangsu China; 2 Orthopaedic Institute, Medical College, Soochow University, No. 178 East Ganjiang Road, Suzhou, 215000 Jiangsu China; 3 Department of Pathology, The Third Affiliated Hospital of Soochow University, Changzhou, 213003 China

**Keywords:** Arctiin, Osteoarthritis, Extracellular matrix, NRF2, Microcarrier

## Abstract

Loss of extracellular matrix (ECM) of cartilage due to oxidative stress injury is one of the main characteristics of osteoarthritis (OA). As a bioactive molecule derived from the traditional Chinese *Burdock*, arctiin exerts robust antioxidant properties to modulate redox balance. However, the potential therapeutic effects of arctiin on OA and the underlying mechanisms involved are still unknown. Based on the Traditional Chinese Medicine Systems Pharmacology Database and Analysis Platform (TCMSP) tool, *Burdock*-extracted small molecule arctiin was identified as a potential anti-arthritic component. In vitro*,* treatment using arctiin rescued the interleukin (IL)-1β-induced activation of proteinases and promoted the cartilage ECM synthesis in human chondrocytes. In vivo*,* intraperitoneal injection of arctiin ameliorated cartilage erosion and encountered subchondral bone sclerosis in the post-traumatic OA mice. Transcriptome sequencing uncovered that arctiin-enhanced cartilage matrix deposition was associated with restricted oxidative stress. Mechanistically, inhibition of nuclear factor erythroid 2-related factor 2 (NRF2) abolished arctiin-mediated anti-oxidative and anti-arthritic functions. To further broaden the application prospects, a gellan gum (GG)-based bioactive gel (GG-CD@ARC) encapsulated with arctiin was made to achieve long-term and sustained drug release. Intra-articular injection of GG-CD@ARC counteracted cartilage degeneration in the severe (12 weeks) OA mice model. These findings indicate that arctiin may be a promising anti-arthritic agent. Furthermore, GG-modified bioactive glue loaded with arctiin provides a unique strategy for treating moderate to severe OA.

**Graphical Abstract:**

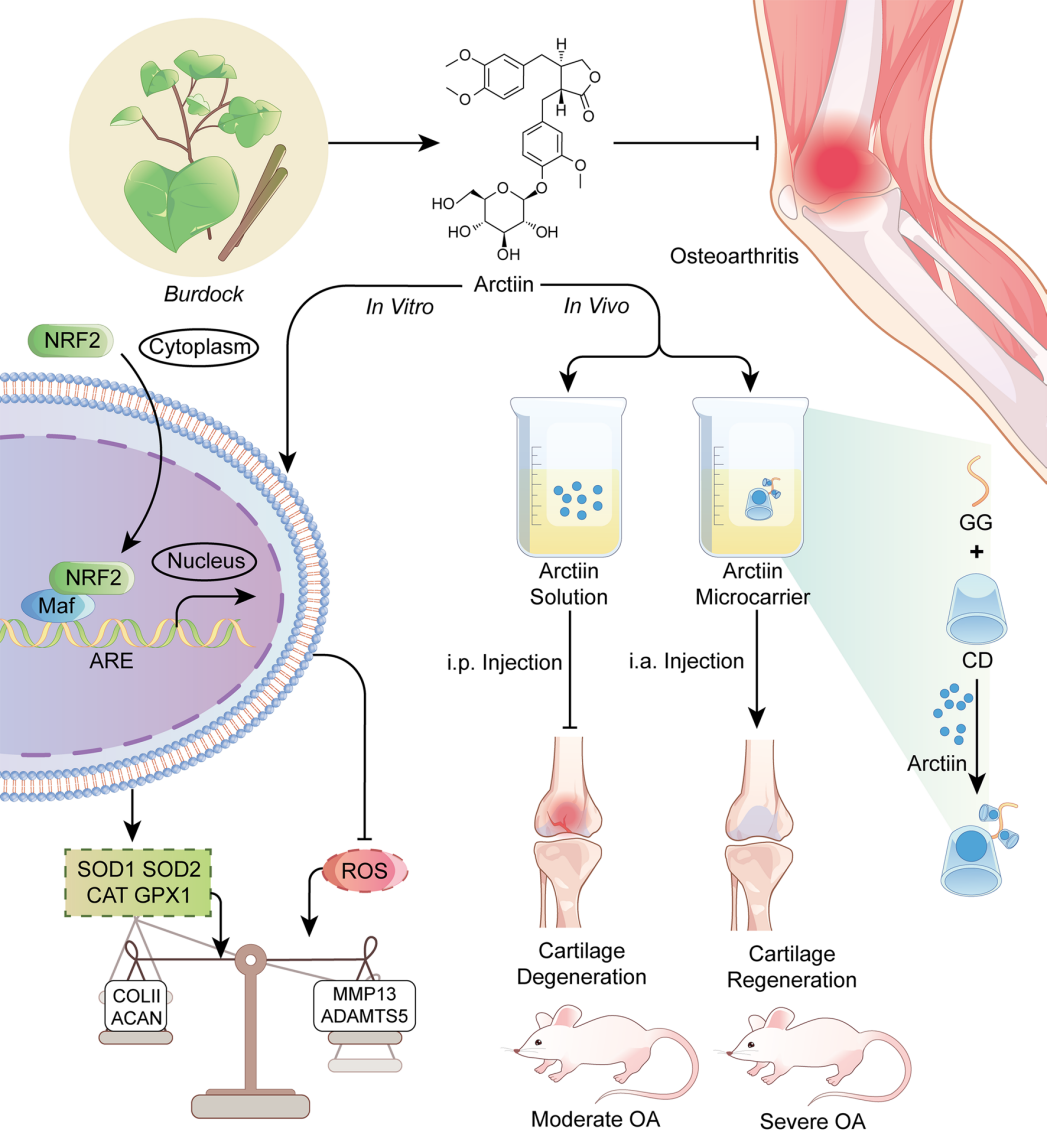

**Supplementary Information:**

The online version contains supplementary material available at 10.1186/s12951-022-01505-7.

## Introduction

Osteoarthritis (OA) is a burdensome degenerative disease with the rapidly aging population. It is estimated that 7% of the global population (almost 500 million) suffers from OA, and the number of patients increased by 48% in the past 30 years. [1] In China, the prevalence of knee osteoarthritis (KOA) increased threefold in the last decade, and the crude prevalence is 13.2% for people aged 50 and over [2]. The hallmark of OA pathogenesis is the metabolic disturbance of the extracellular matrix (ECM). This accelerates the progression of cartilage degeneration by inhibiting matrix synthetic proteins and activating matrix-degrading enzymes [3]. Abnormal ECM catabolism is associated with redox imbalance. As a result, there is an overproduction of reactive oxygen species (ROS), such as superoxide anion, hydrogen peroxide and nitric oxide [4]. Meanwhile, intracellular antioxidant enzymes, including superoxide dismutase (SOD), catalase (CAT), and glutathione peroxidase (GPX) etc., are responsible for maintaining redox homeostasis by scavenging hazardous ROS that are abundant in OA [5]. Hence, further aggravating the oxidative stress injury in chondrocytes [6]. In order to prevent OA-induced cartilage degradation, the restoration of redox balance by preserving chondrocyte antioxidant functions is a promising strategy.

Traditional Chinese Medicine (TCM) has been used in the management of several intractable skeletal disorders. Fructus Arctii, a dried ripe fruit of Arctium lappa L. (*Burdock*, family Asteraceae), is listed in the Chinese pharmacopeia as a well-known Chinese Materia Medica [7]. Arctiin (ARC) is an essential lignan isolated from *Burdock* and has promising effects on cardiac hypertrophy [8], acute lung injury [9], and ischemia/reperfusion diseases [10]. Chen et al. recently reported that arctiin treatment inhibits in vitro osteoclastogenesis and attenuates in vivo bone loss by inhibiting the activation of nuclear factor-κB ligand (RANKL)-induced mitogen-activated protein kinase (MAPK) pathway [11]. In addition, arctiin attenuates silicosis-induced lung injury by maintaining the mitochondrial redox equilibrium and inhibiting the activation of nucleotide-binding oligomerization domain (NOD)-like receptor family protein 3 (NLRP3) inflammasome. Coincidently, arctiin regulates the secretion of transforming growth factor-β (TGF-β), which acts as an enhancer for the chondrogenic differentiation of mesenchymal stem cells (MSCs) [12]. However, the protective effects of arctiin on cartilage ECM and the redox balance mechanisms remain unknown.

Nuclear factor E2-related factor 2 (NRF2), an oxidation-sensitive transcription factor, is involved in regulating oxidative stress via a specific nuclear-translocation process. It increases the binding of the antioxidant response element (ARE) and activates the transcription of over 200 antioxidant enzymes (GST) [13]. In vivo, knockout of *Nrf2* aggravates both the monosodium iodoacetate (MIA) and the destabilization of the medial meniscus (DMM)-induced mice OA model [14]. Activation of NRF2, induced by natural ingredients such as allicin, sulforaphane, and lycopene, attenuates hydrogen peroxide (H_2_O_2_)-mediated oxidative stress and cartilage matrix degradation in vitro [15]. The intra-articular injection of *Nrf2*-overexpressed lentiviral vector ameliorates articular cartilage degradation by suppressing chitinase 3-like-1 (CHI3L1)-mediated inflammation [16]. Our previous study indicated that kartogenin (KGN) enhances the translational expression of NRF2, prevents matrix degradation enzyme activity such as matrix metalloproteinase (MMP)-13 and a disintegrin metalloproteinase with thrombospondin motifs (ADAMTS)-5, and delays OA progression in vivo [17]. Zhou et al. demonstrated that administration of arctiin prevents the H9N2 avian influenza virus. The viral-induced inflammation is down-regulated through NRF2-c-Jun N-terminal kinase (JNK)-MAPK axis [18]. However, the biochemical interaction between arctiin and NRF2 is still unknown in OA.

Gellan gum (GG) is an exopolysaccharide derived from the fermentation process of *Sphingomonas elodea* bacteria [19]. As a product of natural origin, GG possesses excellent biocompatibility and multiple biological properties. The US Food and Drug Administration (FDA) has approved GG as a food additive, and several GG-related commercial products have been developed, e.g., Gelzan^®^, Gel-Gro^®^, and GELRITE^®^ [20]. As a versatile injectable with good cell affinity, GG is considered as an advent in tissue engineering particularly in cartilage and bone regeneration [21]. In relation to the presence of a backbone glucuronic acid residue, the structure of GG resembles that of the natural glycosaminoglycan (GAG) component of the articular cartilage [22]. Biofunctional GG matrices support chondrocyte viability and promote chondrogenic differentiation, thereby promoting the formation of new cartilage via the hyaline-like ECM. Furthermore, in vivo*,* implantation of the cell-laden methacrylate-modified GG (GGMA) hydrogel promoted a better de novo cartilage regeneration in a rabbit focal chondral lesion model [23]. Therefore, injectable cell or drug-laden bio-functional GG may be a potential remedy for cartilage degeneration.

This study aimed to investigate the in vitro protective function of arctiin on cartilage matrix metabolism and validate the in vivo therapeutic effects of systemic administration of arctiin on the post-traumatic OA model. On a biochemical level, the involvement of NRF2 in the anti-arthritic effect of arctiin has been determined. Ultimately, a novel GG-based antioxidant microcarrier was perfected to facilitate the sustained release of arctiin in severe OA.

## Materials and methods

### Bioinformatic prediction

Traditional Chinese Medicine Systems Pharmacology Database and Analysis Platform (TCMSP) was utilized to search for the potential bioactive ingredients of Burdock. The online databases, including Genecards and Online Mendelian Inheritance in Man (OMIM), were chosen to identify the targets of OA. A medicine-ingredients-targets-disease (M-I-T-D) model was established using CytoScape software, and protein–protein interactions (PPI) were illustrated by STRING software. Gene Ontology (GO) and Kyoto Encyclopaedia of Genes and Genome (KEGG) enrichment analyses were performed with R.

### Isolation and culture of human chondrocytes

The study protocol was done following the guidelines and approved by the Ethics Committee of the First Affiliated Hospital of Soochow University. Informed consent was obtained from all patients. Cartilage samples were collected from six randomly selected knee OA patients (4 females and 2 males, aged 58.1 ± 12.3) who underwent total knee arthroplasty (TKA). Articular cartilage was separated from the femoral condyle and the tibial plateau. The cartilage was then digested with 0.2% type II collagenase (Sigma-Aldrich, St. Louis, MO, USA) at 37 °C overnight. The primary chondrocytes were cultured in Dulbecco’s modified Eagle’s medium: nutrient mixture F-12 (DMEM/F-12, Thermo Fisher Scientific, Waltham, MA, USA) supplemented with 10% fetal bovine serum (FBS, Thermo Fisher Scientific), penicillin (100 units/mL) and streptomycin (100 units/mL) at 37 °C in an incubator at 5% CO_2_. The subsequent experiments were performed on chondrocytes from passage one.

### Cell treatment

To mimic an in vitro arthritic microenvironment, chondrocytes were incubated with 10 ng/mL human recombinant IL-1β for the indicated period. Arctiin (Topscience, Shanghai, China) was dissolved in dimethylsulfoxide (DMSO, Sigma-Aldrich) and diluted in a complete culture medium at a gradient concentration of 2.5 μM, 5 μM, or 10 μM. The cells were treated with a selective NRF2 inhibitor, ML385, to inhibit the NRF2 activity (5 μM, Topscience).

### Cell proliferation

The cell proliferation rate was determined using the Cell Counting Kit-8 assay (Beyotime Institute of Biotechnology, Haimen, China) following the manufacturer’s instructions. Chondrocytes were seeded in a 96-well plate at an initial density of 1000 per cm^2^ and treated with 2.5 μM, 5 μM, and 10 μM arctiin. On day 1, 3, 5, and 7, the cells were incubated with 10% CCK8 solution at 37 °C for 1 h. The absorbance value was measured at 450 nm using a microplate reader (BioTek, Winooski, VT, USA).

### Immunofluorescence

Chondrocytes were fixed in cold methanol at room temperature for 15 min and then 0.25% Triton X-100 (Sigma-Aldrich) was added for 15 min. Cells were incubated with the specific anti-COLII (Abcam, Cambridge, MA, USA, ab34712, 1:100), anti-MMP13 (Proteintech, Wuhan, China, 18165-1-AP, 1:50), anti-ACAN, or anti-ADAMTS5 primary antibodies at 4 °C overnight. After washing twice with PBS, the cells were incubated with goat anti-rabbit IgG (H&L) (ab150079) at room temperature for 2 h. The nuclei were counterstained with DAPI (Thermo Fisher Scientific) for 1 min, and the immunofluorescence images were visualized using a Zeiss Axiovert 40CFL microscope (Zeiss, Oberkochen, Germany).

### ROS measurement

The cells were dissociated with 0.25% trypsin–EDTA and centrifuged at 12,000 g for 5 min. The cell suspension was incubated with a 10 μM DCFH-DA (Beyotime) or 10 μM mitoSOX (Thermo Fisher Scientific) solution at 37 °C for 15 min. After washing with PBS three times, the fluorescence intensity was measured using a Guava easycyte Flow Cytometer (Millipore, Boston, MA, USA). 10,000 cells from each sample were then analyzed using the FlowJo 10 software (TreeStar, San Carlos, CA, USA). The cells were incubated with mitoSOX for 15 min and observed using a Zeiss Axiovert 40CFL microscope for mitochondrial ROS detection.

### Quantitative real-time reverse transcription-polymerase chain reaction (RT-PCR)

Total RNA was extracted using a TRIzol^®^ reagent (Thermo Fisher Scientific) and reverse-transcribed to cDNA using a RevertAid First Strand cDNA Synthesis Kit (Thermo Fisher Scientific). Following the manufacturer’s instructions, quantitative real-time reverse transcription-polymerase chain reaction (RT-PCR) was performed using an iTap™ Universal SYBR^®^ Green Supermix kit (Bio-Rad, Hercules, CA, USA) under a CFX96™ Real-Time PCR System (Bio-Rad). The transcript levels of *Col2a1, Acan, Mmp13, Adamts5, Sod1, Sod2, Cat, Gpx1,* and *Nrf2* were evaluated with *Gapdh* as an internal control. The relative expression of mRNAs was calculated using the ΔCt (2^−ΔΔCt^) method. The primer sequences are shown in Additional file 2: Table S1.

### Western blot assay

Total proteins were extracted from chondrocytes using a cell lysis buffer (Beyotime) supplemented with proteinase inhibitor on ice for 1 h. The protein concentrations were then measured using a BCA protein assay kit (Beyotime). The extracted protein (10 μg per sample) was subjected to a 10% sodium dodecyl sulfate–polyacrylamide gel (SDS-PAGE) and then transferred electrophoretically onto a nitrocellulose membrane (Beyotime). The membranes were then blocked in a blocking buffer for 30 min and incubated with primary antibodies against COLII (ab188570), ACAN (A11691), MMP13 (ab39012), ADAMTS5 (ab41037), SOD1 (10269-1-AP), SOD2 (ab13533), CAT (ab209211), GPX1 (ab108427), NRF2 (ab137550), and α-tubulin (AF5012) at a temperature of 4 °C overnight. Subsequently, the membranes were incubated with horseradish peroxidase (HRP)-conjugated anti-mouse or anti-rabbit secondary antibodies for 1 h at room temperature. Bound antibodies were incubated with a SuperSignal West Pico Substrate (Thermo Fisher Scientific) and imaged on a VersaDocTM imaging system (Bio-Rad). The intensity of the bands was quantified using the Image J software (National Institutes of Health, Bethesda, MD, USA), and the relative protein levels were calculated as the ratio of specific band optical intensity to α-tubulin.

### RNA sequencing

Gene expression profiles were measured by Affymetrix Human HTA2.0 expression microarrays (Affymetrix, Santa Clara, CA, USA). Chondrocytes seeded on 6-well plates were treated with 10 μM arctiin. Subsequently, the total RNA was extracted with TRIzol^®^ reagent and quantified using the NanoDrop ND-2000 (Thermo Fisher Scientific) following the manufacturer’s protocol. The RNA sequencing experiments were performed by the Shanghai OE Biotech. Co., Ltd. (Shanghai, China). The gene expression levels between the control (CTRL) and the arctiin (AC) group were compared with the Significant Analysis of Microarray software (SAM), and enrichment analysis for the GO and KEGG were performed using WebGestalt. The differentially expressed genes (DEG) were screened by a fold-value change of ≥ 1.5 in the SAM output results. The differently expressed genes (DEGs) of CTRL or arctiin-treated chondrocytes were then analyzed using Gene set enrichment analysis (GSEA) software (http://www.broad.mit.edu/GSEA, v.4.0.3), based on online platforms including Reactome gene sets and KEGG pathways.

### Synthesis of GG-CD@ARC carrier

Low-acyl gellan gum (GG, Macklin, Shanghai, China, 1%) was dissolved in a 50 mM 2-(N-Morpholino) ethanesulfonic acid hydrate (MES) buffer and stirred at 90 °C for 2 h. Then, 1-ethyl-3-(3-dimethylaminopropyl) carbodiimide (EDC, Macklin) and N-hydroxysulfosuccinimide (sulfo-NHS, Macklin) were added in GG solution and stirred at 60 °C for 30 min. The 6-(6-aminohexyl) amino-6-deoxy-β-cyclodextrin (CD, Macklin) was added to the GG solution and underwent the carbodiimide reaction at 60 °C overnight. The product was dialyzed in ddH_2_O for 3 days and then lyophilized for 48 h. In order to manufacture the GG-CD@ARC carrier, 1 mg/mL of arctiin was dissolved in 1% GG-CD solution at 60 °C for 2 h.

### Characterization of GG-CD@ARC

#### Scanning electron microscope (SEM) analysis

In an effort to observe the ultrastructure of the GG-CD@ARC carrier, the lyophilized samples were sputtered with a thin Au layer on the surface. The carrier size and the surface pores were scanned using an SEM (Quanta 250, USA) at a voltage of 10 kV.

#### Fourier transform infrared (FTIR) spectroscopy

Lastly, to confirm the specific functional groups, GG, CD, and GG-CD samples were pressed into potassium bromide (KBr) pellets. The tablets were then subjected to an FTIR spectrometer (Thermo Fisher, Nicolet 6700, USA) with a wavelength ranging from 400 to 4000 cm^−1^ using 128 scans at 4 cm^−1^ resolution.

#### X-ray diffraction (XRD) measurement

X-ray powder diffraction (XRD) was performed using an X-ray diffractometer (Bruker D8 Advance, Germany) at 40 kV and 40 mA to monitor the crystalline nature of the inclusion complex. Data were analyzed from a *2θ* value between 10° and 40° under CuKα radiation.

#### Release profile

As a means to detect the arctiin release, 10 mg of arctiin was added into 20 mL of GG (1 mg mL^−1^) or GG-CD (1 mg mL^−1^) solution. The mixture was dialyzed in 30 mL of distilled water and shaken at a temperature of 37 °C. 15 mL of dialysate was collected, and 15 mL of fresh distilled water was changed. The cumulative release rate of arctiin was calculated using a Nanodrop 2000 spectrometer (Thermo Fisher Scientific) at a wavelength of 450 nm.

#### Biocompatibility test

Chondrocytes were seeded in the 96-well or 24-well plates and treated with GG-CD@ARC leachate to evaluate the biocompatibility of GG-CD@ARC carrier on cells. In the cell proliferation assay, cells were treated with 10% CCK8 solution and observed using a 450 nm wavelength microplate reader. To evaluate the cytotoxicity, cells were incubated with live/dead reagent (Thermo Fisher Scientific) at a temperature of 37 °C for 15 min. The live (green) or dead (red) cells were captured using a Zeiss Axiovert 40CFL microscope.

### DMM-induced mice OA

The animal protocols were approved by the Ethics Committee of Soochow University (SUDA20211227A01). Seven-week-old C57BL/6J mice (male) were purchased from the Experimental Animal Center of Soochow University. In order to induce a post-traumatic OA model, the DMM surgery was performed on C57BL/6J mice under anesthesia using sodium pentobarbital. The knee joint capsules were exposed, and the medial meniscotibial ligament (MMTL) was transected using a micro scissor. In contrast, the knee joint capsules were incised without ligament resection in the Sham group. To avoid potential infection, mice were injected with 10,000 U of penicillin intramuscularly for three consecutive days postoperatively.

### Animal treatment

Arctiin was initially dissolved in DMSO and then diluted in 0.9% saline for intraperitoneal or intra-articular injection. One week after surgery, 10 μL of saline, arctiin (10 mg/kg), GG-CD (1 mg/mL), or GG-CD@ARC (1 mg/mL) was delivered intraperitoneally or intra-articularly in the knee joints. Mice were randomly separated into four groups of six mice for the first animal experiment: sham, sham + ARC, DMM, DMM + ARC. Injections of saline or arctiin were performed twice a week intraperitoneally.

In the second experiment, mice were divided into four groups, each with six mice: DMM, DMM + ARC, DMM + GG-CD, and DMM + GG-CD@ARC. Injections of saline, arctiin, GG-CD, or GG-CD@ARC were performed once a week intra-articularly. Mice were euthanized at eight or twelve weeks post-operatively, and the intact knee joints were harvested for subsequent analyses.

### μCT analysis

The subchondral bone sclerosis was assessed using a micro-computed tomography (μCT) system (Skyscan 1176, Kontich, Belgium) with a high resolution (9 μm) at 50 kV (200 μA). Data reconstruction was conducted with NRecon v1.6 and CTAn v1.13.8.1 software. Regions of interest (ROI) were defined as the 30 consecutive layers of the medial tibial subchondral bone. The subchondral bone-specific parameters include bone volume ratio (BV/TV, %), trabecular thickness (Tb.Th, mm), and trabecular separation (Tb.Sp., mm^−1^).

### Histology and immunohistochemistry (IHC)

The knee samples were fixed in 10% formalin for 48 h and decalcified in 10% ethylene diamine tetraacetic acid (EDTA) buffer (Shanghai Yuanye Bio-Technology Co., Ltd., Shanghai, China) for three weeks. Samples were dehydrated with gradient ethanol and then embedded in paraffin (Thermo Fisher Scientific). Sagittal sections of a thickness of 6 μm were prepared using a rotary microtome (Leica, Weztlar, Germany). To examine the morphology and proteoglycans of the cartilage, the sections were stained with Safranin-O/fast green and hematoxylin and eosin (H&E) (Sigma-Aldrich). The level of cartilage erosion was quantified using the Osteoarthritis Research Society International (OARSI) scoring system, and the cartilage thickness was calculated using the ratio of hyaline cartilage to fibrous cartilage. For immunohistochemical staining, the sections were incubated with 1% hydrogen peroxide (Sigma-Aldrich) for 30 min and retrieved using 2 mg/mL testicular hyaluronidase (Sigma-Aldrich) for 1 h. After blocking with 1.5% goat serum, the slides were incubated with anti-COLII (ab188570) and anti-NRF2 (ab137550) primary antibodies at 4 °C overnight. The slides were then incubated with a second antibody (Vector Laboratories, Burlingame, CA, USA) at room temperature for 1 h. Subsequently, the slides were stained with 3,3′-Diaminobenzidine (DAB, Vector Laboratories, Burlingame, CA, USA). Images were captured under a bright-field microscope (Zeiss Axiovert 200, Oberkochen, Germany).

### Statistical analysis

Data are reported as the mean ± standard deviation (SD). Statistical analyses were performed using a two-tailed Student’s *t*-test between two groups or one-way Analysis of Variance (ANOVA) with Tukey’s post hoc test among multiple comparisons, using the SPSS 13.0 statistical software (SPSS Inc., Chicago, IL, USA). A *p-value* < 0.05 was considered statistically significant.

## Results

### Bioinformatic analysis between Burdock and OA

**Fig. 1:**
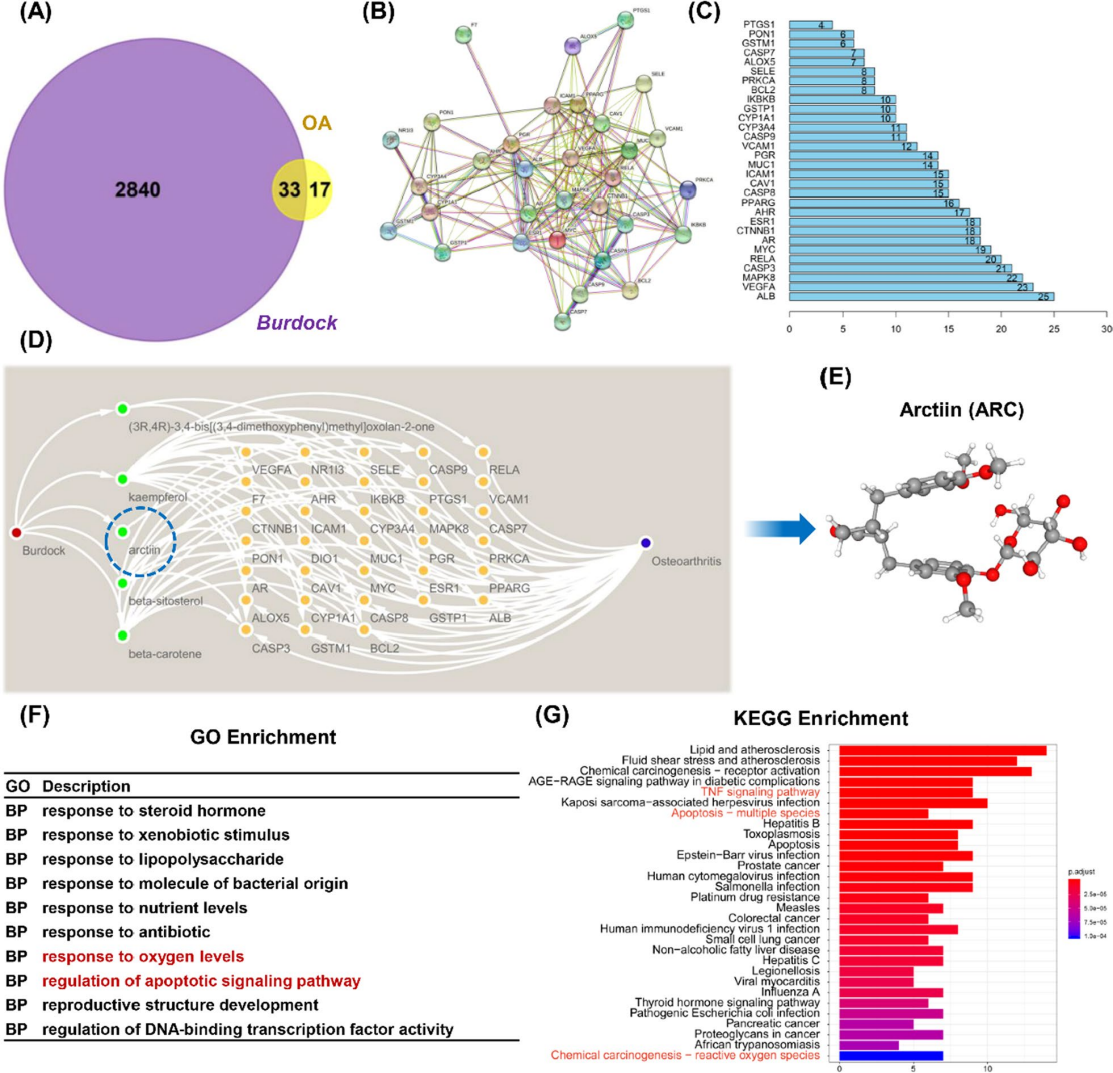
Integrated bioinformatics analysis between Chinese herb *Burdock* and OA. **A** The intersected gene targets between the medicine (*Burdock*) and the disease (OA). **B** Proteins and proteins interaction network of the 33 identified targets. **C** The rank of potential key proteins according to the nodes number. **D** Medicine-Ingredient-Target-Disease (M-I-T-D) network model. **E** The structure of the *Burdock* extract, arctiin (ARC). **F** GO enrichment analysis based on the differentially expressed genes (DEGs). **G** KEGG pathways analysis in accordance with DEGs

Arctiin is a plant lignan extracted from *Burdock* seeds, with a molecular weight (MW) of 534.6. The Venn map showed 33 intersected target genes between *Burdock* and OA (Fig. 1A). PPI indicated that albumin (ALB), vascular endothelial growth factor A (VEGFA), and MAPK8 are potential targets (Fig. 1B, C). The Medicine-Ingredient-Targets-Drug (MITD) model indicated that arctiin, β-sitosterol, kaempferol, β-carotene, and (3R,4R)-3,4-bis[(3,4-dimethoxyphenyl) methyl] oxolan-2-one regulate OA progression by targeting 33 potential sites (Fig. 1D). These are potential action sites for arctiin (Fig. 1E). According to the differentially expressed genes (DEGs), response to oxygen levels and regulation of apoptotic signaling pathway were identified as pivotal BP components (Fig. 1F). KEGG analysis revealed that apoptosis, tumor necrosis factor (TNF) signaling pathway and reactive oxygen species (ROS) were screened as three of the top 25 pathways (Fig. 1G).

### Arctiin maintains ECM metabolic balance in in vitro arthritic environment

**Fig. 2:**
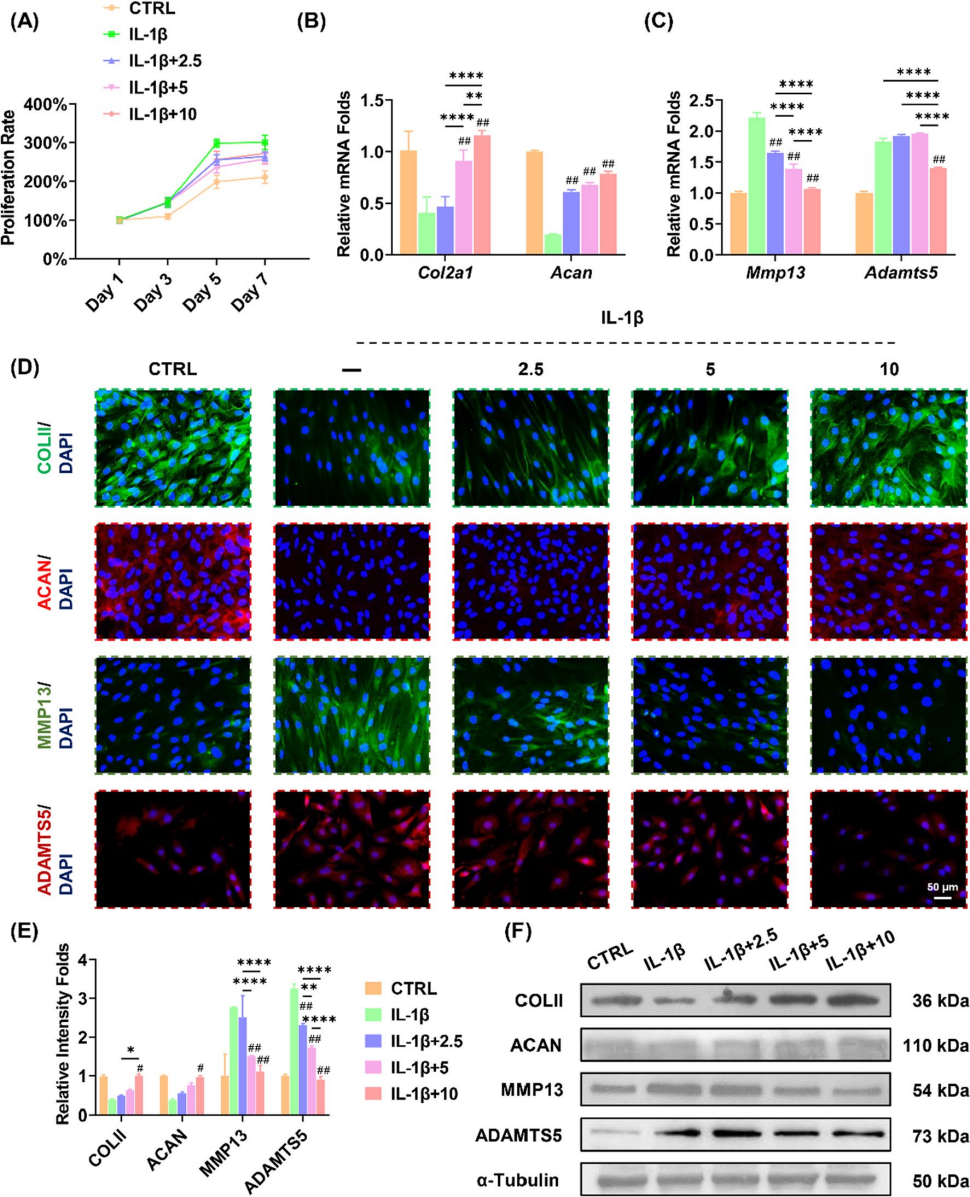
In vitro arctiin treatment regulates cartilage extracellular matrix (ECM) homeostasis. **A** The cell viability of arctiin-treated chondrocytes at 2.5, 5, or 10 μM concentration following stimulation with 10 ng/mL IL-1β (n = 6). **B** The transcript levels of anabolic genes, including *Col2a1* and *Acan,* were quantified with real-time RT-PCR using *GAPDH* as the internal reference (n = 4). **C** The transcript levels of the catabolic genes *Mmp13* and *Adamts5* were quantified with real-time RT-PCR (n = 4). **D**, **E** Immunofluorescence staining indicated the expression of ECM synthesis markers: COLII and ACAN, and the ECM degrading enzymes: MMP13 and ADAMTS5 (n = 3). **F** The protein levels of COLII, ACAN, MMP13, and ADAMTS5 were determined using Western blot assays (n = 3). Values represent mean ± SD. Statistically, significant differences are indicated by ^#^ where *p* < 0.05, ^##^ where *p* < 0.01 compared with the IL-1β group or * where *p* < 0.05, ** where *p* < 0.01 between the indicated groups

The effects of arctiin on cell proliferation and ECM metabolism of chondrocytes were then investigated. Human chondrocytes (passage one) were treated with arctiin at 2.5, 5, or 10 μM in the presence of IL-1β (10 ng/mL). The CCK-8 assays indicated that IL-1β increased the pathological cell proliferation by 42.5% compared to the CTRL group at day 7. Simultaneously, acrtiin inhibited pathological hyper-proliferation in chondrocytes (Fig. 2A). The RT-PCR results demonstrated that 10 μM of arctiin up-regulated the gene expression of *Col2a1* and *Acan* by 1.8-fold and 3.0-fold, respectively, in comparison to the IL-1β group (Fig. 2B). Furthermore, 10 μM of arctiin down-regulated the transcript levels of proteinases (*Mmp13* by 52.0% and *Adamts5* by 23.5%) (Fig. 2C). The immunofluorescence staining showed that cartilage ECM equilibrium was disrupted in IL-1β-stimulated chondrocytes. Arctiin treatment promoted the matrix synthesis markers but inhibited matrix degradation enzymes in a dose-dependent manner (Fig. 2D, E). Western blot data confirmed that the protein levels of cartilage ECM metabolic balance were maintained by the arctiin treatment (Fig. 2F, Additional file 1: Fig. S1A).

### Systemic administration of arctiin ameliorates cartilage degeneration

**Fig. 3:**
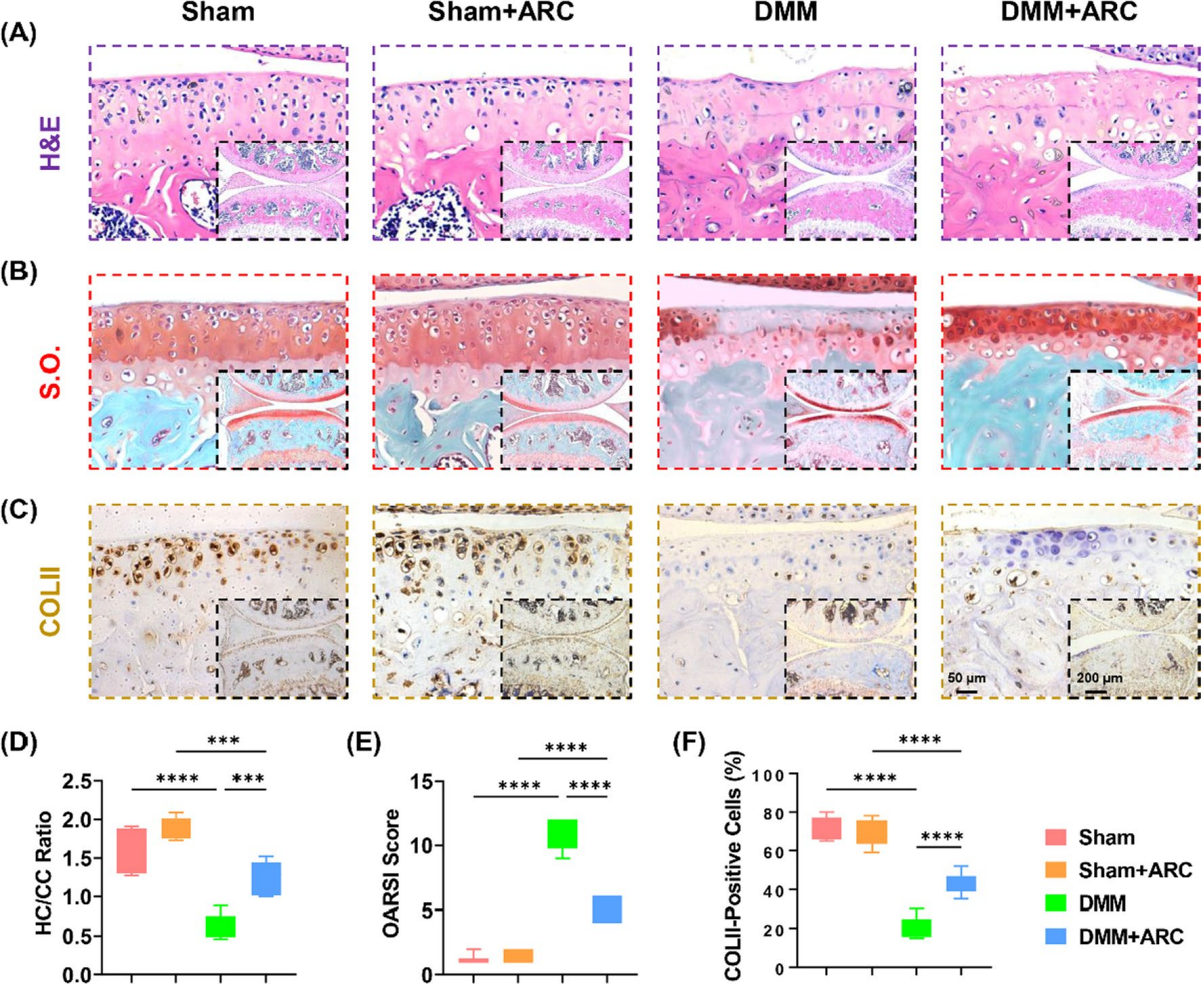
In vivo administration of arctiin mitigated DMM-induced articular cartilage abrasion in seven-week-old C57BL/6J male mice. **A** Representative images of articular cartilage stained by hematoxylin and eosin (H&E). **B** Representative images of articular cartilage stained by safranin O and fast green (S.O.). **C** Representative images of articular cartilage stained by collagen type II (COLII). **D** Quantification of the ratio of hyaline cartilage (HC) versus calcified cartilage (CC) (n = 6). **E** Quantification of the Osteoarthritis Research Society International (OARSI) score (n = 6). **F** Quantification of the percentage of COLII-positive cells in the articular cartilage (n = 6). Values represent the mean ± SD. Statistically significant differences are indicated by * where *p* < 0.05, ** where *p* < 0.01, *** where *p* < 0.001 and **** where *p* < 0.0001 between the indicated groups

To explore the therapeutic effects of arctiin on cartilage degeneration in vivo, C57BL/6J mice were treated with 10 mg/mL arctiin via intraperitoneal injection. Eight weeks after DMM surgery, histological analysis revealed that the administration of arctiin prevented DMM-induced articular cartilage degeneration (Fig. 3A). The ratio of HC/CC in the DMM + arctiin group was 1.0-fold higher than that in the DMM group (Fig. 3D). Cartilage erosion was increased after the DMM surgery but was attenuated in the arctiin-treated group (Fig. 3B), as evidenced by a 50.8% decrease in OARSI score (Fig. 3E). Furthermore, arctiin treatment increased the percentage of COLII-positive cells by 102.4% compared to the DMM group (Fig. 3C, F).

**Fig. 4:**
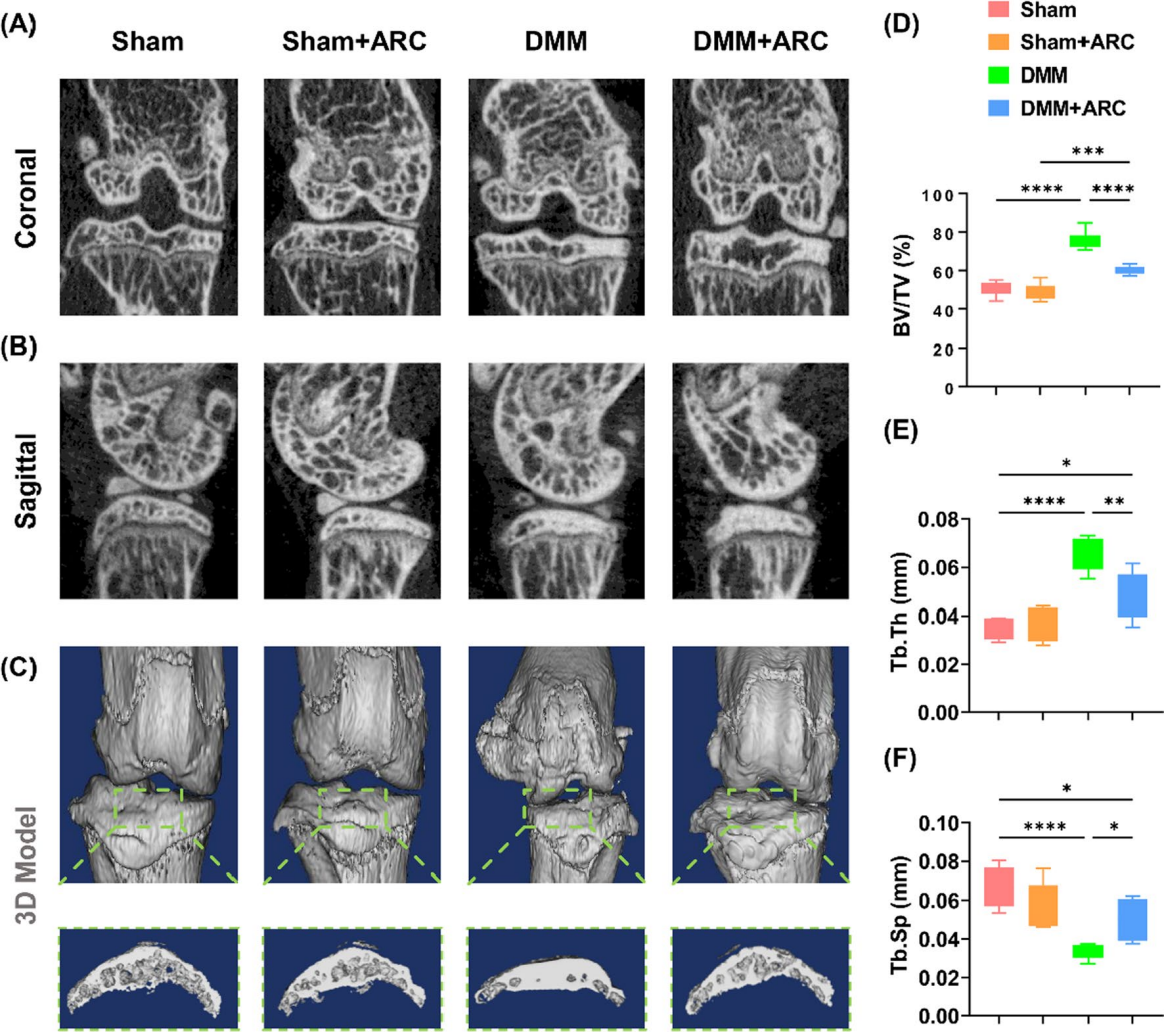
Intraperitoneal injection of arctiin ameliorated DMM-induced subchondral bone sclerosis in seven-week-old C57BL/6J male mice. **A**, **B** 2D reconstructed coronal and sagittal model of the mice knee joints. **C** 3D reconstructed model and the formation of osteocytes of the medial tibial plateau. **D**–**F** The effects of arctiin treatment on the values of bone volume ratio (BV/TV, %), trabecular thickness (Tb.Th, mm), and trabecular separation (Tb.Sp., mm^−1^) (n = 6). Values represent mean ± SD. Statistically significant differences are indicated by * where *p* < 0.05, ** where *p* < 0.01, *** where *p* < 0.001 and **** where *p* < 0.0001 between the indicated groups

μCT showed significantly more severe subchondral bone sclerosis in the post-traumatic mice than in the Sham group, but treatment with arctiin alleviated the abnormal subchondral bone hyperplasia (Fig. 4A, B). 3D reconstruction analysis showed severe osteophyte formation at the medial distal femur and tibial plateau in DMM mice, but the features were less prominent with arctiin treatment (Fig. 4C). Quantitative data indicated that intraperitoneal injection of arctiin resulted in a marked decrease of 20.4% in BV/TV (Fig. 4D), Tb.Th by 27.6% (Fig. 4E), and an increase of 50.4% in Tb.Sp (Fig. 4F).

### Transcriptomics analysis for arctiin-treated chondrocytes

**Fig. 5:**
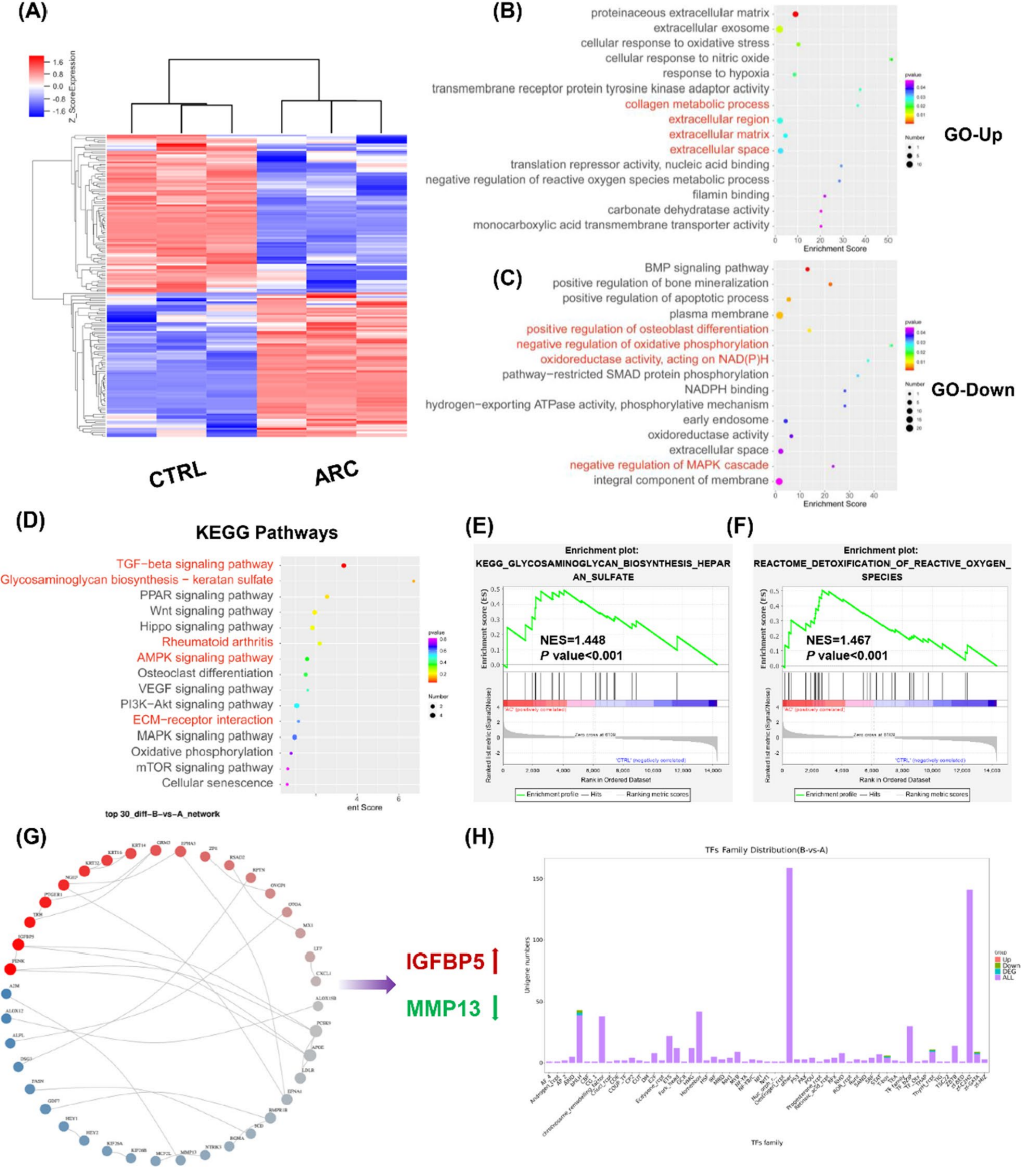
RNA sequencing analysis between CTRL and arctiin-treated chondrocytes. **A** The heat map showed the differentially expressed genes (DEGs) between CTRL and ARC groups (n = 3). **B**, **C** GO enrichment analysis indicated the up-regulated or down-regulated biological processes according to the DEGs. **D** KEGG enrichment analysis revealed the up-regulated or down-regulated pathways. **E**, **F** GSEA enrichment analysis identified the potential KEGG or Reactome pathways. **G** Proteins and proteins interaction (PPI) based on the DEGs. **H** Transcriptional factors (TFs) distribution analysis between CTRL and arctiin-treated group

The whole transcriptome RNA sequencing was performed using CTRL and arctiin-treated chondrocytes. 168 differentially expressed genes (DEGs), including 80 up-regulated genes and 88 down-regulated genes, were screened (Fig. 5A). The GO enrichment analysis revealed an up-regulation of ECM substances such as collagen metabolic process, extracellular region, extracellular matrix, and extracellular space following arctiin treatment (Fig. 5B). Conversely, arctiin treatment down-regulated the oxidative stress and osteogenesis components. For instance, arctiin treatment negatively regulated the osteoblast differentiation and the regulation of MAPK cascade (Fig. 5C). Subsequent KEGG enrichment analysis revealed that arctiin treatment regulated cartilage ECM metabolism via TGF-β signaling pathway, glycosaminoglycan biosynthesis, or AMPK signaling pathway (Fig. 5D). In order to validate the above-mentioned results, proofreading of KEGG and Reactome pathways analysis based on the GESA tools were performed. As demonstrated by enriched findings, the arctiin-regulated biological effects are mediated by GAG biosynthesis and ROS detoxification (Fig. 5E). Following arctiin treatment, protein and protein interaction (PPI) analysis revealed increased insulin like growth factor binding protein 5 (IGFBP5) expression and decreased MMP13 expression. (Fig. 5G). The following transcriptional factors (TFs) enrichment identified crucial upstream modulators such as GATA binding protein 3 (GATA3) and hepatocyte nuclear factor 4 gamma (HNF4G) (Fig. 5H).

### Arctiin protects cartilage homeostasis via NRF2-enhanced antioxidant effects

**Fig. 6:**
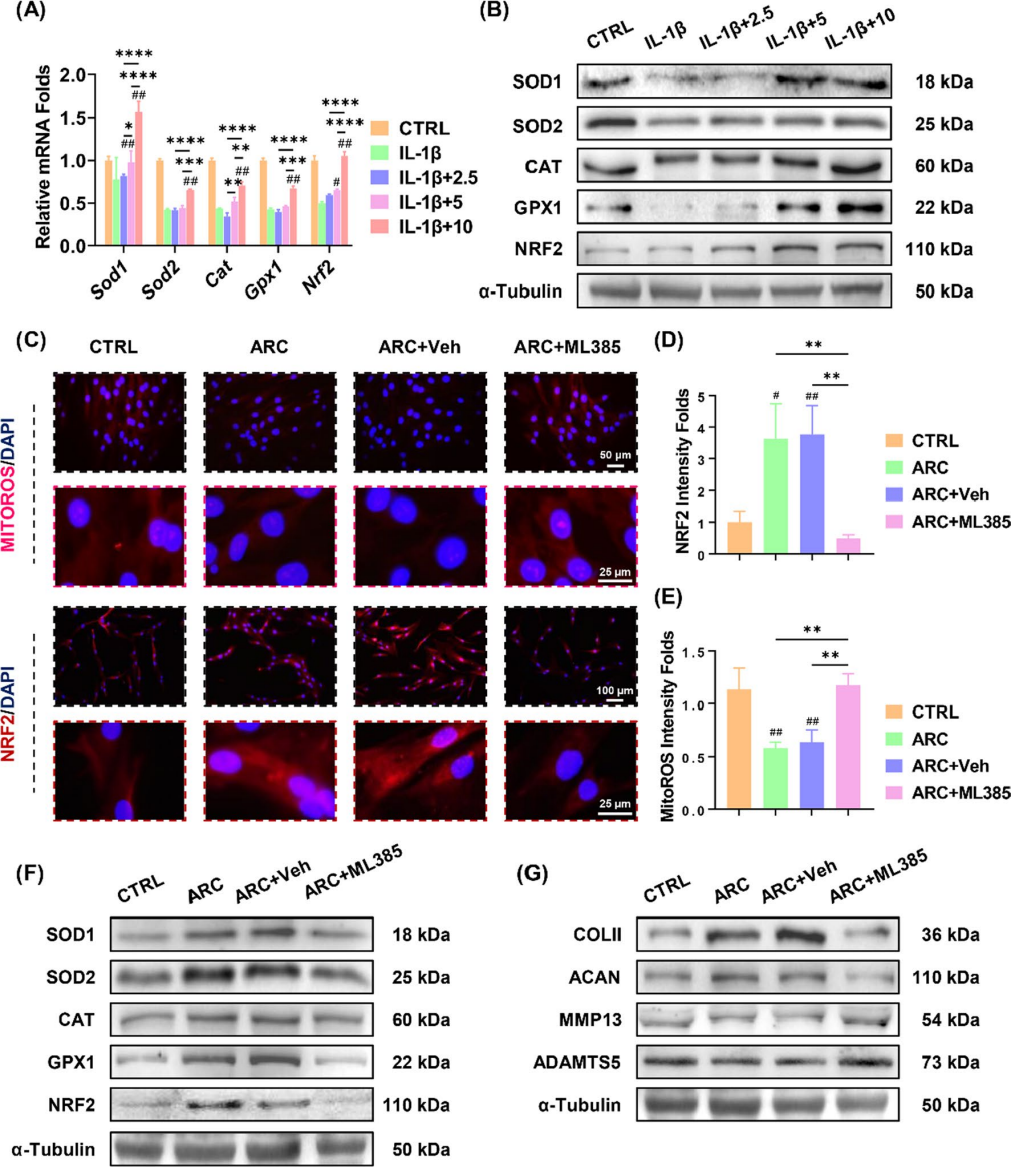
Arctiin protected against OA via activation of NRF2-mediated antioxidant property. **A** The transcript levels of antioxidant enzymes including *Sod1*, *Sod2*, *Cat*, *Gpx1*, and *Nrf2* were quantified with real-time RT-PCR (n = 4). **B** The protein levels of SOD1, SOD2, CAT, GPX1, and NRF2 were determined using Western blot assays (n = 3). **C** Representative images of the immunofluorescence staining of NRF2 or MitoROS. **D**, **E** Quantification of NRF2 activity or Mitochondrial ROS accumulation (n = 3). **F**, **G** The protein levels of antioxidant markers and ECM anabolic markers were determined using Western blot assays (n = 3). Values represent mean ± SD. Statistically, significant differences are indicated by ^#^ where *p* < 0.05, ^##^ where *p* < 0.01 compared with the IL-1β group or * where *p* < 0.05, ** where *p* < 0.01 between the indicated groups

In order to understand the underlying mechanism involving arctiin-induced anti-arthritic effects, the impact of arctiin treatment on redox balance in IL-1β-treated chondrocytes was studied. RT-PCR results indicated that IL-1β impaired the antioxidant enzymes activities; however, arctiin treatment dose-dependently increased the transcript levels of *Sod1*, *Sod2*, *Cat*, and *Gpx1*. (Fig. 6A). In the presence of 10 μM of arctiin, the mRNA expression of *Nrf2*, a key antioxidant transcription factor, was increased by 1.1-fold in comparison to the IL-1β group. The protein levels of antioxidant enzymes were also up-regulated by arctiin treatment, as demonstrated by the Western blot experiments (Fig. 6B, Additional file 1: Fig. S1B). Furthermore, the antioxidant properties inhibited the accumulation of intracellular or mitochondrial ROS caused by IL-1β (Additional file 1: Fig. S2A-B).

Taken together with the bioinformatic findings of response to oxygen levels and RNA sequencing results of ROS detoxification, arctiin-exerted chondroprotective effects may depend on regulation of redox balance. Since its dominant status in modulating oxidative homeostasis, NRF2-associated anti-arthritic effects was first prioritized. Chondrocytes were treated with ML385 (an NRF2-specific inhibitor) to suppress NRF2 activity. The immunofluorescence staining revealed that treatment with arctiin increased the nuclear translocation of NRF2, whereas such effect was abolished by the ML385 treatment (Fig. 6C, D). Moreover, the NRF2-dependent antioxidant system suppressed abnormally produced mitochondrial ROS (Fig. 6E). RT-PCR data showed that ML385 decreased the mRNA expression of *Sod1* by 31.3%, *Sod2* by 52.5%, *Cat* by 33.0%, *Gpx1* by 50.6%, and *Nrf2* by 75.3% (Additional file 1: Fig. S3A). Western blot analysis confirmed that ML385 treatment had a detrimental effect on intracellular antioxidant enzymes (Fig. 6F, Additional file 1: Fig. S3B). Additionally, the presence of ML385 altered the transcript levels of *Col2a1*, *Acan*, *Mmp13*, and *Adamts5* (Additional file 1: Fig. S3C). The protein levels of cartilage ECM markers were confirmed by Western blot assays (Fig. 6G, Additional file 1: Fig. S3D).

### GG-CD@ARC achieves sustained release and good biocompatibility

**Fig. 7:**
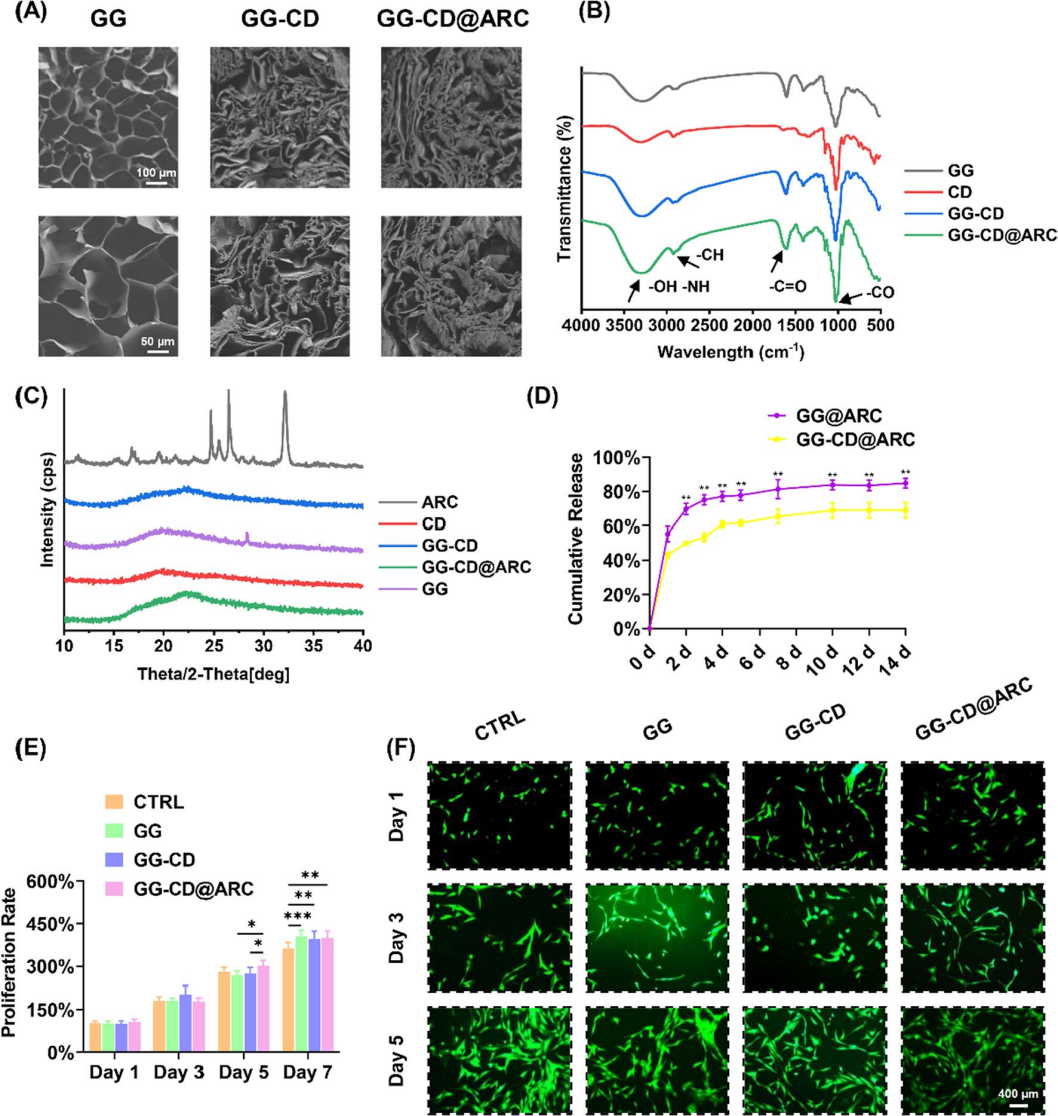
Characteristics of the fabricated GG-CD@ARC biological glue. **A** Representative images of the surface morphology using a scanning electron microscope (SEM). **B** Fourier transform infrared (FTIR) showed a successful grafting reaction between gellan gum and cyclodextrin. **C** The crystal form of composites was detected using X-ray powder diffraction (XRD). **D** The cumulative release curves of arctiin at each 2-week time-point (n = 6). **E** The impact of each complex on cell proliferation was quantified using the CCK-8 method (n = 6). **F** The viability of cells cultured with leachate and stained with Live/Dead assays (n = 3). Values represent mean ± SD. Statistically significant differences are indicated by * where *p* < 0.05, ** where *p* < 0.01, *** where *p* < 0.001 and **** where *p* < 0.0001 between the indicated groups

SEM showed the continuous porous ultrastructure of GG, GG-CD, or GG-CD@ARC and the attached arctiin particles on the surface of GG-CD were observed (Fig. 7A). FTIR spectroscopy showed a reaction occurring in the GG-CD carboxyl group following the alteration of the C = O and CO peaks at 1620 cm^−1^ and 1040 cm^−1^, respectively (Fig. 7B). The XRD analysis indicated that arctiin’s crystalline structure peaked at a 2*θ* = 24.8°, 26.7°, and 32.4°. However, GG-CD@ARC yielded an amorphous character without the formation of crystalline peaks. The results confirm the formation of an inclusion complex (Fig. 7C). As for the cumulative release profiles, GG-CD@ARC achieved a better extended release of arctiin within two weeks compared to the non-inclusion group (Fig. 7D). Chondrocytes were cultured with the GG-CD@ARC-derived leachate for 7 days in order to evaluate the biocompatibility of GG-CD@ARC in vitro. The improved cell proliferation in GG or modified GG-derived leachate (11.7% higher in the GG group, 9.4% higher in the GG-CD group, and 9.4% higher in GG-CD@ARC group) was determined in the CCK-8 experiment (Fig. 7E). Furthermore, the live/dead assay concluded that GG, GG-CD, or GG-CD@ARC did not produce cytotoxic effects on chondrocytes (Fig. 7F, Additional file 1: Fig. S4A-C).

### Intra-articular injection of GG-CD@ARC promotes cartilage regeneration

**Fig. 8:**
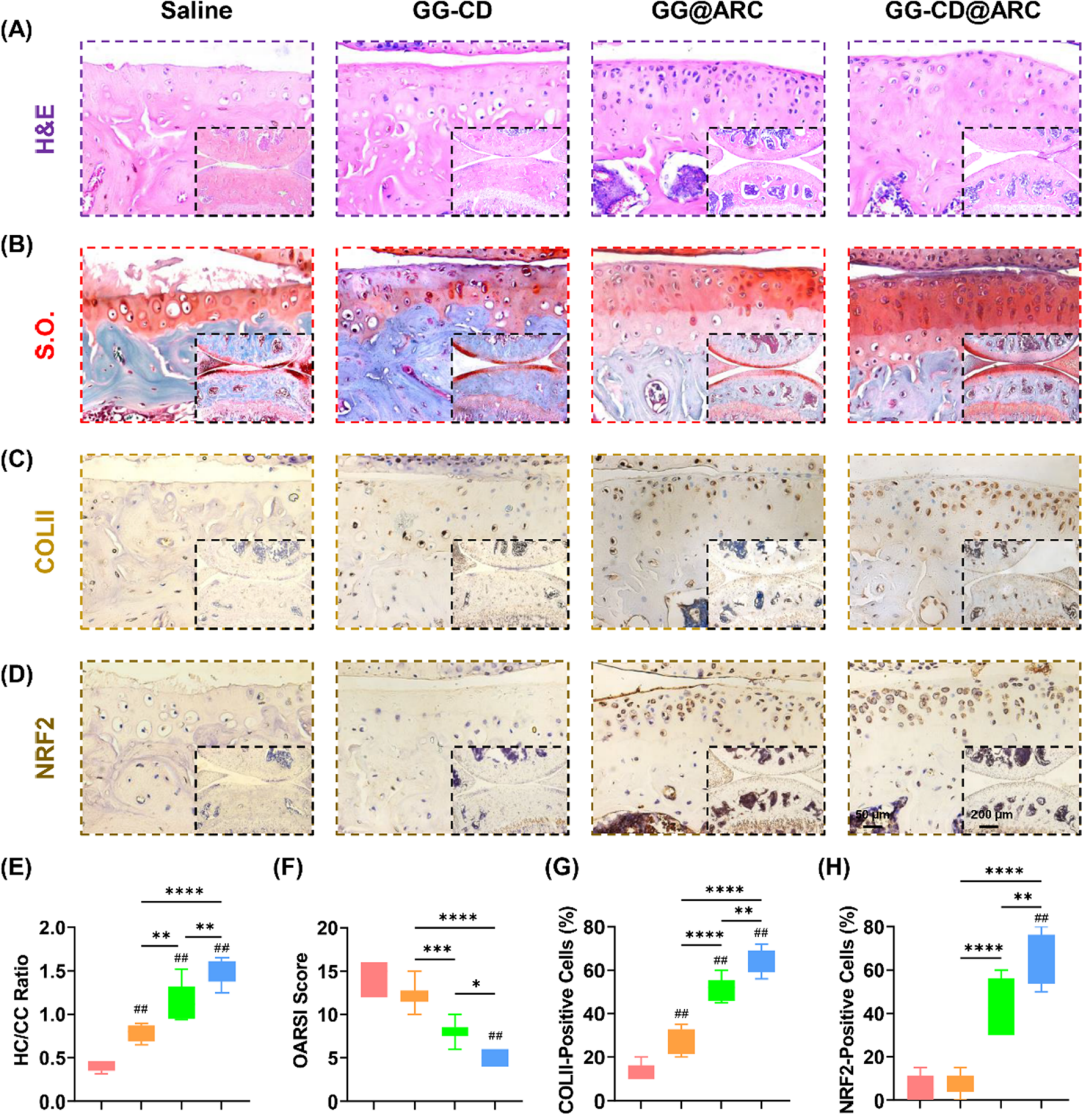
In vivo administration of GG-CD@ARC rectified DMM-induced articular cartilage abrasion in seven-week-old C57BL/6J male mice. **A** Representative images of articular cartilage stained by hematoxylin and eosin (H&E). **B** Representative images of articular cartilage stained by safranin O and fast green (S.O.). **C** Representative images of articular cartilage stained by collagen type II (COLII). **D** Representative images of articular cartilage stained by NRF2. **E** Quantification of the ratio of hyaline cartilage (HC) versus calcified cartilage (CC) (n = 6). **F** Quantification of the Osteoarthritis Research Society International (OARSI) score (n = 6). **G** Quantification of the percentage of COLII-positive cells in the articular cartilage (n = 6). **H** Quantification of the percentage of NRF2-positive cells in the articular cartilage (n = 6). Values represent the mean ± SD. Statistically significant differences are indicated by * where *p* < 0.05, ** where *p* < 0.01, *** where *p* < 0.001 and **** where *p* < 0.0001 between the indicated groups

In order to assess the clinical application of arctiin, GG-CD@ARC microcarrier was delivered into the knee joint via intra-articular injection to evaluate the long-term therapeutic effects. Twelve weeks after DMM surgery, the sulfated GAGs in the post-traumatic OA model were remarkedly preserved with GG-CD@ARC (Fig. 8A, B). The HC/CC ratio and OARSI score in the GG-CD@ARC-treated group were improved by 2.6-fold (Fig. 8E) and 65.1%, respectively (Fig. 8F) as opposed to the saline group. The intra-articular injection treatment with GG-CD@ARC increased the percentage of COLII-positive by 3.7-fold (Fig. 8C, G) and NRF2-positive cells by 10.1-fold (Fig. 8D, H).

**Fig. 9:**
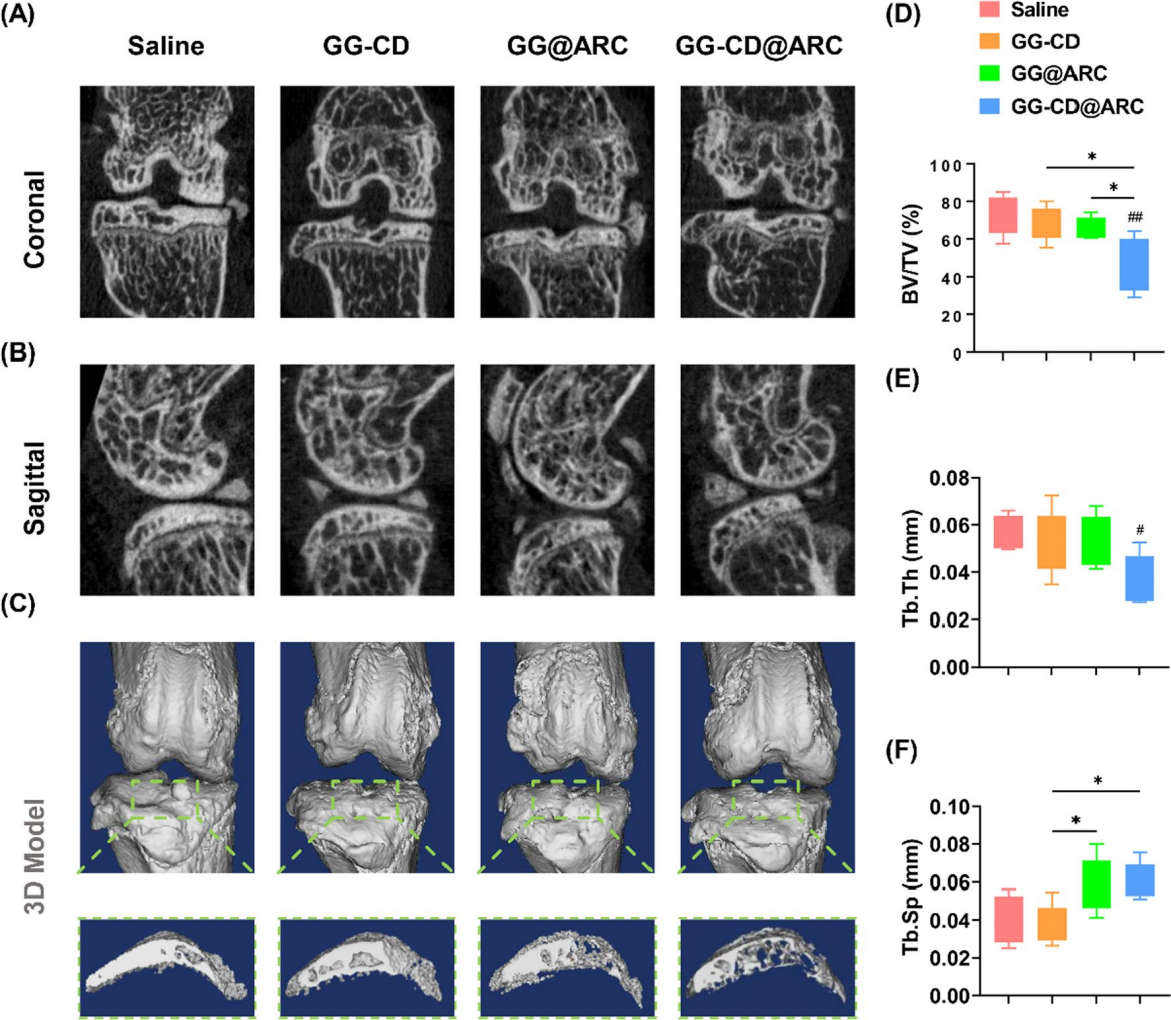
Intra-articular injection of GG-CD@ARC attenuated subchondral bone sclerosis in a severe experimental OA model in seven-week-old C57BL/6J male mice. **A**, **B** 2D reconstructed coronal and sagittal models of the mice knee joints. **C** 3D reconstructed model and the formation of osteocytes on the medial tibial plateau. **D**–**F** The effect of arctiin treatment on the values of bone volume ratio (BV/TV, %), trabecular thickness (Tb.Th, mm), and trabecular separation (Tb.Sp., mm^−1^) (n = 6). Values represent mean ± SD. Statistically, significant differences are indicated by * where *p* < 0.05, ** where *p* < 0.01, *** where *p* < 0.001 and **** where *p* < 0.0001 between the indicated groups

Moreover, the three-month OA model also showed a significant level of abnormal bone formation in the deep zone of the subchondral bone. In contrast, treatment with GG-CD@ARC prevented DMM-induced subchondral bone sclerosis (Fig. 9A, B) and osteophyte formation (Fig. 9C). Quantitative analysis showed that treatment with GG-CD@ARC improved the BV/TV by 37.5% (Fig. 9D), Tb.Th by 34.5% (Fig. 9E), and Tb.Sp by 51.2% (Fig. 9F) compared to the saline group.

## Discussion

TCM, as a component of the traditional medical system, possesses a long and rich history in many Asian countries. In regards to degenerative musculoskeletal disorders, e.g., OA, osteoporosis (OP) [24], and intervertebral disc degeneration (IVDD) [25], the beneficial effects of classical Chinese herbal formulae have been fully established. Clinically, a randomized controlled trial (RCT) found that oral administration of *Burdock* root tea (1.5 g) three times per day decreased the recurrence rate of acute colonic diverticulitis (ACD) by 20% as compared to the control group [26]. Additionally, daily consumption of *Burdock* root tea (6 g) decreased serum inflammatory cytokines such as interleukin-6 (IL-6), C-reactive protein (CRP), and malondialdehyde, while increasing SOD activity to alleviate oxidative stress in patients with KOA [27]. However, the complex ingredients in TCM formulae obstruct research into the mechanism of action and limit clinical application. Currently, novel strategies using bioinformatic tools, such as network pharmacology of TCM (TCMNP) and system pharmacology of TCM (TCMSP), help to identify the key bioactive constituents and hence reveal the underlying mechanism of *Burdock* [28]. Given the beneficial analgesic effect of the *Burdock root* for OA patients, a network pharmacological analysis between *Burdock* and OA was performed to unveil its mode of action. The established M-I-T-D network model indicated that arctiin (a lignan derived from *Burdock*) is implicated in the *Burdock*-mediated anti-arthritic effects. In the animal model, intraperitoneal injection of 40 mg/kg arctiin once daily for eight consecutive weeks attenuates albuminuria and glomerulosclerosis in diabetic nephropathy (DN) in rats [29]. For the first time, the results demonstrated that arctiin prevented IL-1β-mediated cartilage ECM loss. Hence, it improves the resistance of chondrocytes to the proinflammatory stimuli of the in vitro arthritic environment. Therefore, arctiin regulates the cartilage ECM metabolic balance by enhancing matrix synthesis capacity and suppressing matrix proteases activity.

Oral intake of *Burdock* tea is the most preferred and convenient route of administration for patients. The monomeric arctiin, intragastric or intraperitoneal administration are the most common in vivo approaches [30]. In line with previous studies, for the first-stage animal experiment, 10 mg/kg of arctiin was injected intraperitoneally into sham or DMM-induced OA mice. Arctiin treatment not only alleviated aberrant subchondral bone sclerosis and osteophyte formation, but also improved articular cartilage integrity by increasing type II collagen and glycosaminoglycan (GAG) expression. Due to its systemic administration, arctiin’s anti-arthritic effect, particularly on vessel-rich subchondral bone, may be proportional to the body’s inflammatory responses and redox balance. Numerous evidences have shown the crosstalk between osteoclast or osteoblast cells and type H vessels synergistically facilitate subchondral angiogenesis and aggravate bone remodeling [31]. Arctiin could protect against high glucose (HG)-induced cell injury in both rat aortic endothelial cell [32] and human retinal capillary endothelium [33]. Given that arctiin alleviated abnormal bone formation in vivo, we inferred that arctiin may negatively regulate the extension of type H vessels and requires further substantiation. Recent studies have suggested that arctiin may serve as a potential anti-osteoporosis (OP) agent via recoupling osteogenic differentiation and osteoclastogenesis. Liu et al. reported that in vitro treatment with arctiin promoted osteogenesis in MC3T3-E1 osteoblastic cells through targeting cyclin D1 pathway [34]. However, different from the continuous bone loss in OP, dynamic alteration of bone remodeling is involved in OA pathogenesis, of which enhanced subchondral bone turnover at early OA but subchondral bone sclerosis during the advanced or late stages [35]. Therefore, the underlying mechanisms of arctiin on osteogenesis will be explored in our future work.

MAPK and phosphatidylinositol 3-kinase (PI3K)-protein kinase B (AKT) were identified by the bioinformatic analysis as the key pathways involved in the crosstalk between arctiin and OA. Cumulative evidence indicates that abnormal activation of p38-MAPK accelerates articular cartilage degeneration [36]. Due to the thorough inhibition of arctiin on MAPK or PI3K/AKT [33], the robust cartilage protection observed in vivo may be mediated in part by these signaling pathways. On the other hand, the signal transducer and activator of transcription 3 (STAT3) is a key component of the STATs protein superfamily and is involved in regulating the inflammatory reaction [37]. The expression level of STAT3 is increased dramatically in the OA-isolated chondrocytes, indicating that the phosphorylation of STAT3 may be involved in the pathogenesis [38]. Moreover, activated STAT3 signaling impacted subchondral bone remodeling by modifying calcium (Ca) and phosphate (Pi) levels in the subchondral milieu [39]. In multiple myeloma (MM), arctiin blocked the STAT3 phosphorylation of tyrosine 705 by activating tyrosine phosphatase ε (PTPε) [40]. IGFBP5, which belongs to the high affinity IGF binding family, is essential for bone remodeling and repair [41]. Local administration of IGFBP5 protein enhanced periodontal tissue regeneration via activation of mesenchymal stem cells’ functions [42]. In medial meniscal tear (MMT)-induced rat OA model, the proteolytic activity towards IGFPB-5 was increased and the IGFBP-5 metabolism was disrupted; however, intra-articular injection of protease inhibitor peptide PB-145 could prevent cartilage erosion and promote regeneration [43]. Moreover, IGFBP5 is positively regulated by mTORC1 [44], which itself is a known modulator in protecting cartilage metabolism and antagonizing OA progression [45]. Our further experiments will discover the involvement of IGFBP5 in arctiin-medicated anti-arthritic effects.

Excess ROS degrades cartilage ECM components by affecting protease activity. Chondrocytes with deficient antioxidant elements are unable to resist negative stimuli. In this regard, arctiin acts as a robust antioxidant to correct the redox imbalance and antagonize cartilage degeneration [46, 47]. According to our RNA sequencing results, oxidative stress injury was relieved by arctiin-treated chondrocytes and was associated with improved matrix synthesis. Owing to the fact that NRF2 act as a critical oxidation-sensitive transcription factor; meanwhile, our recent studies systematically presented the role of NRF2 in promoting bone [48] and cartilage repair [49], thereby NRF2-mediated redox balance in OA protection was further corroborated. The results established that arctiin treatment inhibits IL-1β-induced ROS accumulation in both the cytoplasm and mitochondria by increasing the activity of multiple antioxidative enzymes, including SODs, catalase, and glutathione peroxidase. Biochemically, the inhibition of NRF2 activity nullifies the protective effect of arctiin on oxidative stress and ECM homeostasis. In accord with the above-mentioned results, the latest study found that arctiin antagonizes triptolide (TP)-induced hepatotoxicity. The effect is mediated by antioxidant elements enhanced with NRF2, including heme oxygenase-1 (HO-1) and NAD(P)H quinone oxidoreductase 1 (NQO1) [50]. However, the underlying mechanism by which arctiin regulates NRF2 has not been determined yet. Bae et al. reported that arctiin rescues hydrogen peroxide (H_2_O_2_)-induced cell senescence in human dermal papilla cells by modulating the expression of various microRNAs (miRNAs), such as miR-125a-5p. The latter was up-regulated by fivefold after arctiin treatment [51]. Similarly, miR-125a-5p-abundant exosomes derived from MSCs prevent ECM loss in human chondrocytes by targeting E2F transcription factor 2 (E2F2) [52]. Furthermore, epigenetic modification is implicated in NRF2’s functioning. Kawai et al. illustrated that the transcriptional activity and nucleocytoplasmic localization was affected by its up-stream deacetylase silent mating type information regulation 2 homolog 1 (SIRT1) [53]. Ubiquitin-specific protease 29 (USP29) interacts and deubiquitinates NRF2, thereby regulating NRF2-mediated macrophages polarization in spinal cord injury (SCI) [54]. Therefore, miRNA or epigenetic modification-based regulation may be a potential interaction of arctiin with NRF2.

Since the articular cartilage is an avascular, innervated, and alymphatic substance, systemic administration of arctiin may require a higher concentration, hence resulting in concomitant side effects. Due to the “true-solution” state, inevitable leakage of drugs or molecules occurs frequently in traditional intra-articular drug delivery [55]. To overcome this shortcoming, a gellan gum-based cyclodextrin drug microcarrier was developed for local administration of arctiin. As a temperature-sensitive substance, the solution-state gellan gum at 60 °C transforms into a glue state at 37 °C. This efficient change in state from solution to glue assists in intra-articular injection and allows arctiin to be retained in the joint cavity. In vitro*,* the release profile indicated that the GG-CD@ARC microcarrier could slowly release arctiin within 14 days. GG-CD@ARC was injected into the knee joint and achieved satisfactory repair of severe OA, showing promising biocompatibility. Unlike other acute inflammatory joint diseases, e.g., rheumatoid arthritis (RA), gouty arthritis (GA), or psoriatic arthritis (PA), the development of OA is a gradual and slow process lasting decades. Injection-based therapies require repeated intra-articular administration, which may increase tissue damage and risk of infection [56]. The hydrogel-inspired method effectively prolong the time for the drug to take effect; however, the excessive rigidity of some hydrogels may impair the superlubricity between articular cartilage surfaces [57]. Therefore, a highly viscous “glue-like” microcarrier was introduced to maintain natural mobility and facilitate drug retainment in the articular cavity. In accordance with the findings of the study, dexamethasone-loaded gellan gum alleviates inflammatory response to promote regeneration of cartilage defect in a rabbit model [58]. Recently, it was observed that microfluid microspheres provided lubrication and sustained intra-articular drug release [59]. In our future work, functional microspheres modified with arctiin will be studied to manage OA.

## Conclusions

The study demonstrated that a natural herb-isolated bioactive molecule, arctiin, regulated cartilage ECM metabolism in an in vitro arthritic environment. Systemic administration of arctiin reduced the cartilage erosion and subchondral bone sclerosis in the post-traumatic OA model. Biochemically, the NRF2-dependent oxidative stress balance was involved in the arctiin-induced anti-arthritic effect. Furthermore, the intra-articular injection of the arctiin-loaded microcarrier achieved long-term drug release and prevented cartilage degeneration in severe OA. The antioxidant-enhanced microcarrier discussed in this study provides the tool to implement a novel strategy to counteract OA.

## Supplementary Information

**Additional file 1. Figure S1. (A-B) **The protein levels of COLII, ACAN, MMP13, ADAMTS5, SOD1, SOD2, CAT, GPX1, and NRF2 were determined using Western blot assays.** Figure S2. (A-B) **Intracellular and mitochondrial ROS levels in arctiin-treated chondrocytes were determined using flow cytometry**. Figure S3. (A&C) T**he transcript levels of antioxidant markers: Sod1, Sod2, Cat, Gpx1 and ECM anabolic markers: Col2a1, Acan, Mmp13, and Adamts5 were quantified with real-time RT-PCR. (B&D) Quantification data of SOD1, SOD2, CAT, GPX1, COLII, ACAN, MMP13, and ADAMTS5 were determined using Western blot assays.** Figure S4. (A-C) **Quantification of the viability of cells cultured with leachate and stained with Live/Dead assays at day 1, 3, or 5.

**Additional file 2. Table S1. **Primers used for real-time PCR.

## Data Availability

The data that support the findings of this study are available from the corresponding author upon reasonable request.
