# Supplementary material for: Arctiin-reinforced antioxidant microcarrier antagonizes osteoarthritis progression
Source: J Nanobiotechnology. 2022 Jun 27;20:303. doi: 10.1186/s12951-022-01505-7 (PMC9235181; doi:10.1186/s12951-022-01505-7)
Supplement: Supplementary file 1 — Additional file 1. Figure S1. (A-B) The protein levels of COLII, ACAN, MMP13, ADAMTS5, SOD1, SOD2, CAT, GPX1, and NRF2 were determined using Western blot assays. Figure S2. (A-B) Intracellular and mitochondrial ROS levels in arctiin-treated chondrocytes were determined using flow cytometry. Figure S3. (A&C) The transcript levels of antioxidant markers: Sod1, Sod2, Cat, Gpx1 and ECM anabolic markers: Col2a1, Acan, Mmp13, and Adamts5 were quantified with real-time RT-PCR. (B&D) Quantification data of SOD1, SOD2, CAT, GPX1, COLII, ACAN, MMP13, and ADAMTS5 were determined using Western blot assays. Figure S4. (A-C) Quantification of the viability of cells cultured with leachate and stained with Live/Dead assays at day 1, 3, or 5. [file 12951_2022_1505_MOESM1_ESM.docx]

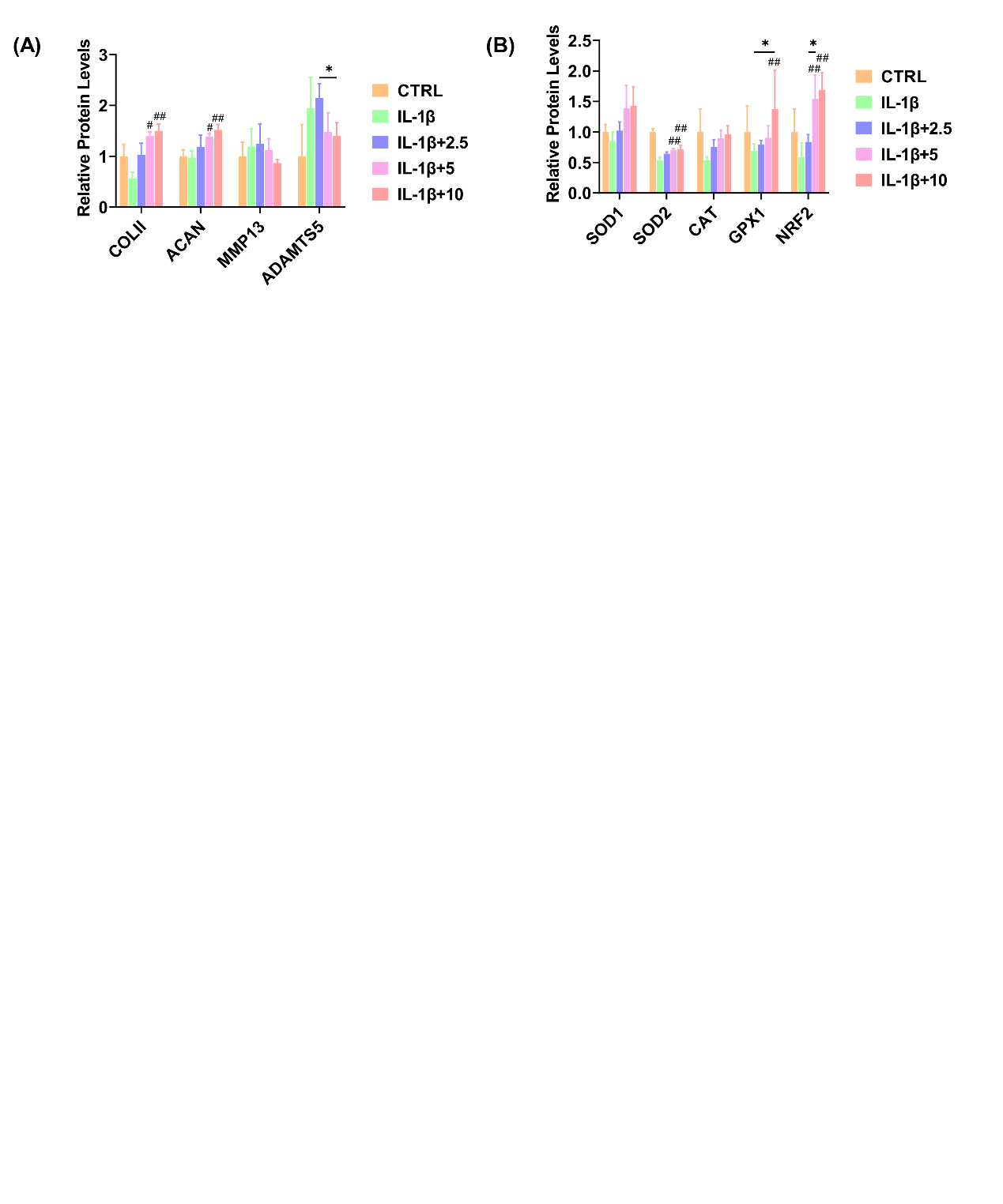


**Supplementary Figure 1.** Arctiin treatment maintains the ECM and redox balance between in IL-1β-treated chondrocytes. The protein levels of COLII, ACAN, MMP13, ADAMTS5, SOD1, SOD2, CAT, GPX1, and NRF2 were determined using Western blot assays (n = 3). Values represent mean ± SD. Statistically, significant differences are indicated by ^#^ where *p* < 0.05, ^##^ where *p* < 0.01 compared with the IL-1β group or * where *p* < 0.05, ** where *p* < 0.01 between the indicated groups.


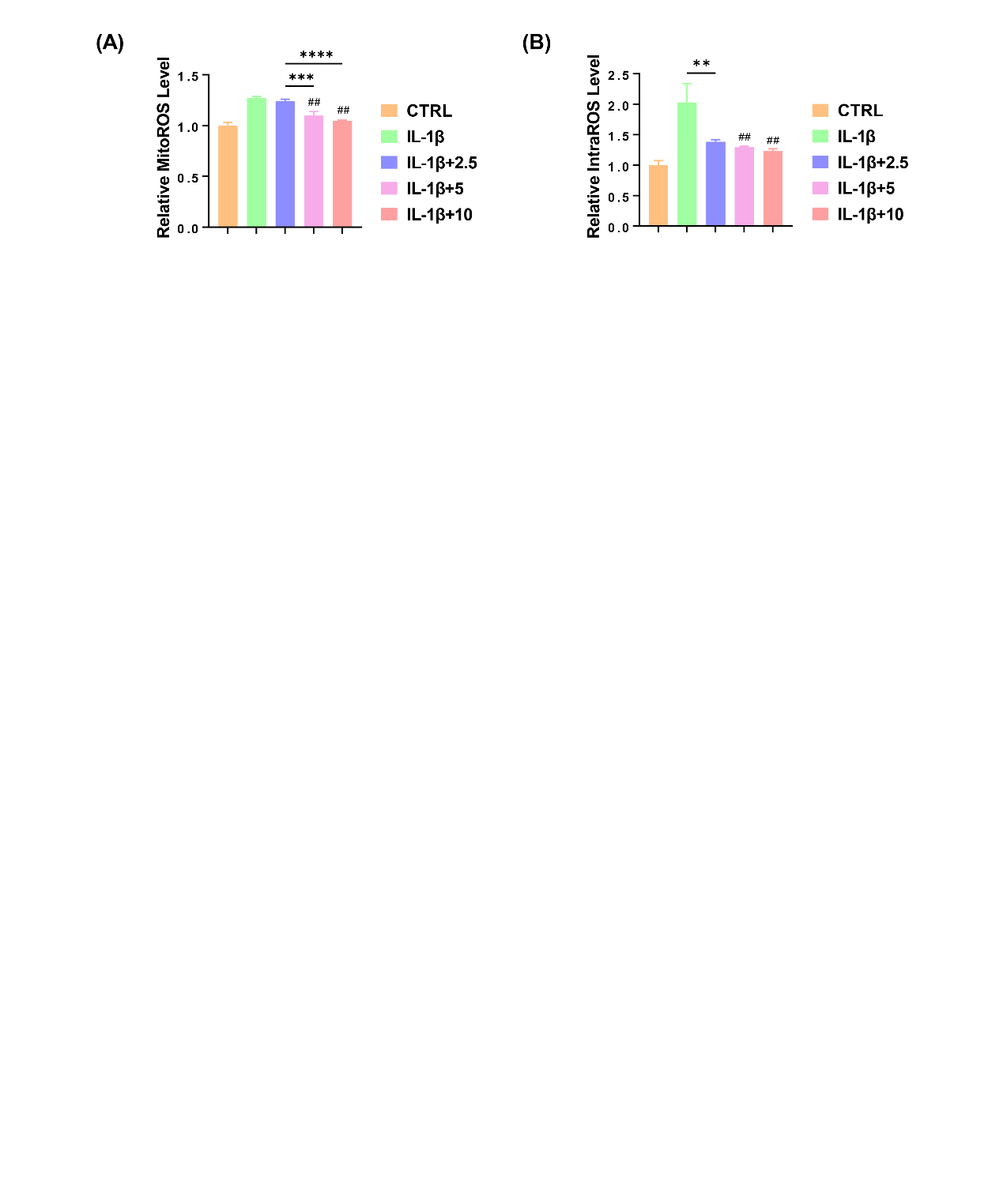


**Supplementary Figure 2.** Arctiin-enhanced antioxidant system scavenges intracellular and mitochondrial ROS levels. (A-B) Intracellular and mitochondrial ROS levels in arctiin-treated chondrocytes were determined using flow cytometry (n = 3). Values represent mean ± SD. Statistically, significant differences are indicated by ^#^ where *p* < 0.05, ^##^ where *p* < 0.01 compared with the IL-1β group or * where *p* < 0.05, ** where *p* < 0.01 between the indicated groups.


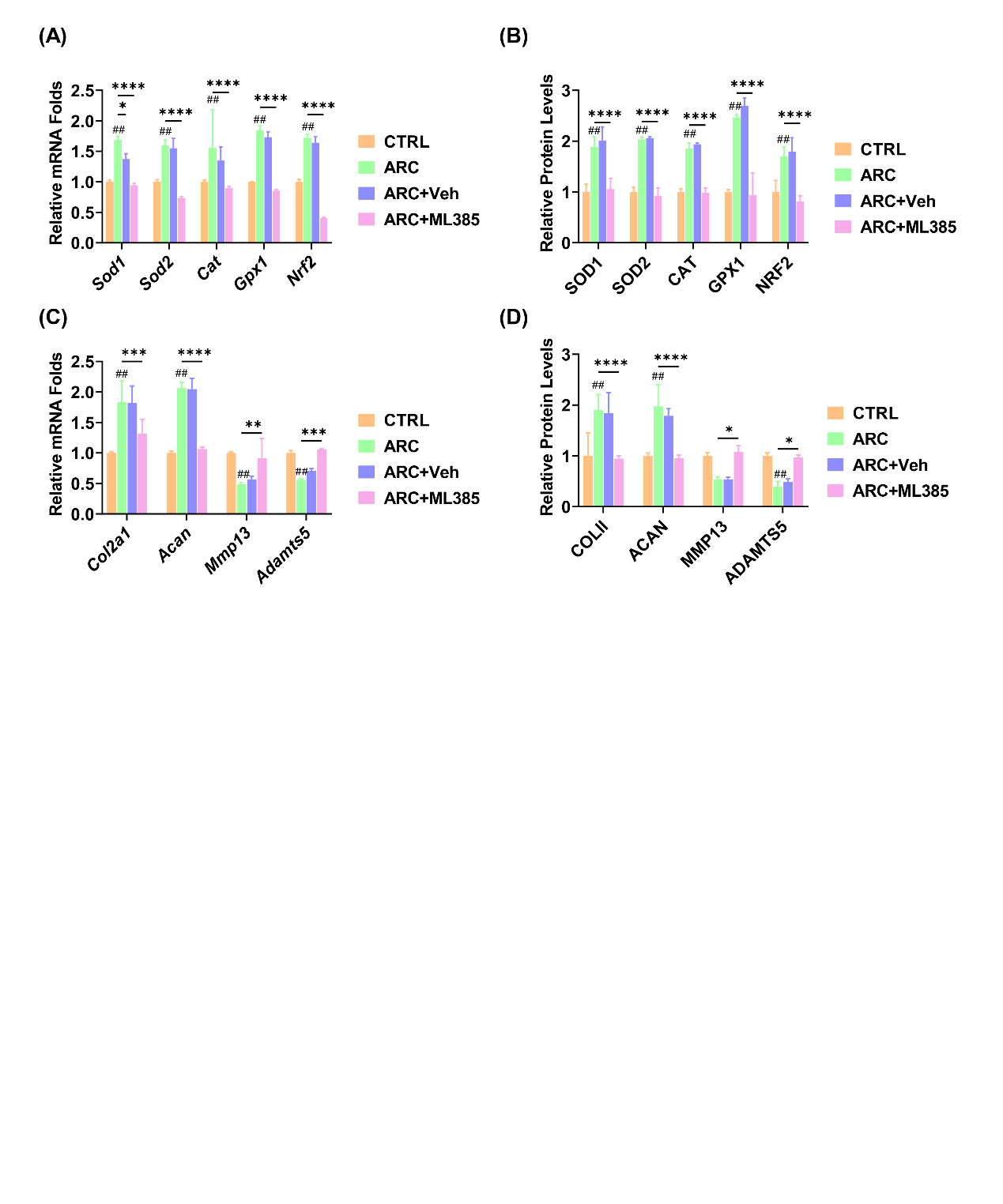


**Supplementary Figure 3.** Inhibition of NRF2 reversed arctiin-mediated cartilage protection via disturbed oxidative stress. (A&C) The transcript levels of antioxidant markers: *Sod1*, *Sod2*, *Cat*, *Gpx1* and ECM anabolic markers: *Col2a1*, *Acan*, *Mmp13*, and *Adamts5* were quantified with real-time RT-PCR (n = 4). (B&D) Quantification data of SOD1, SOD2, CAT, GPX1, COLII, ACAN, MMP13, and ADAMTS5 were determined using Western blot assays (n = 3). Values represent mean ± SD. Statistically, significant differences are indicated by ^#^ where *p* < 0.05, ^##^ where *p* < 0.01 compared with the CTRL group or * where *p* < 0.05, ** where *p* < 0.01 between the indicated groups.


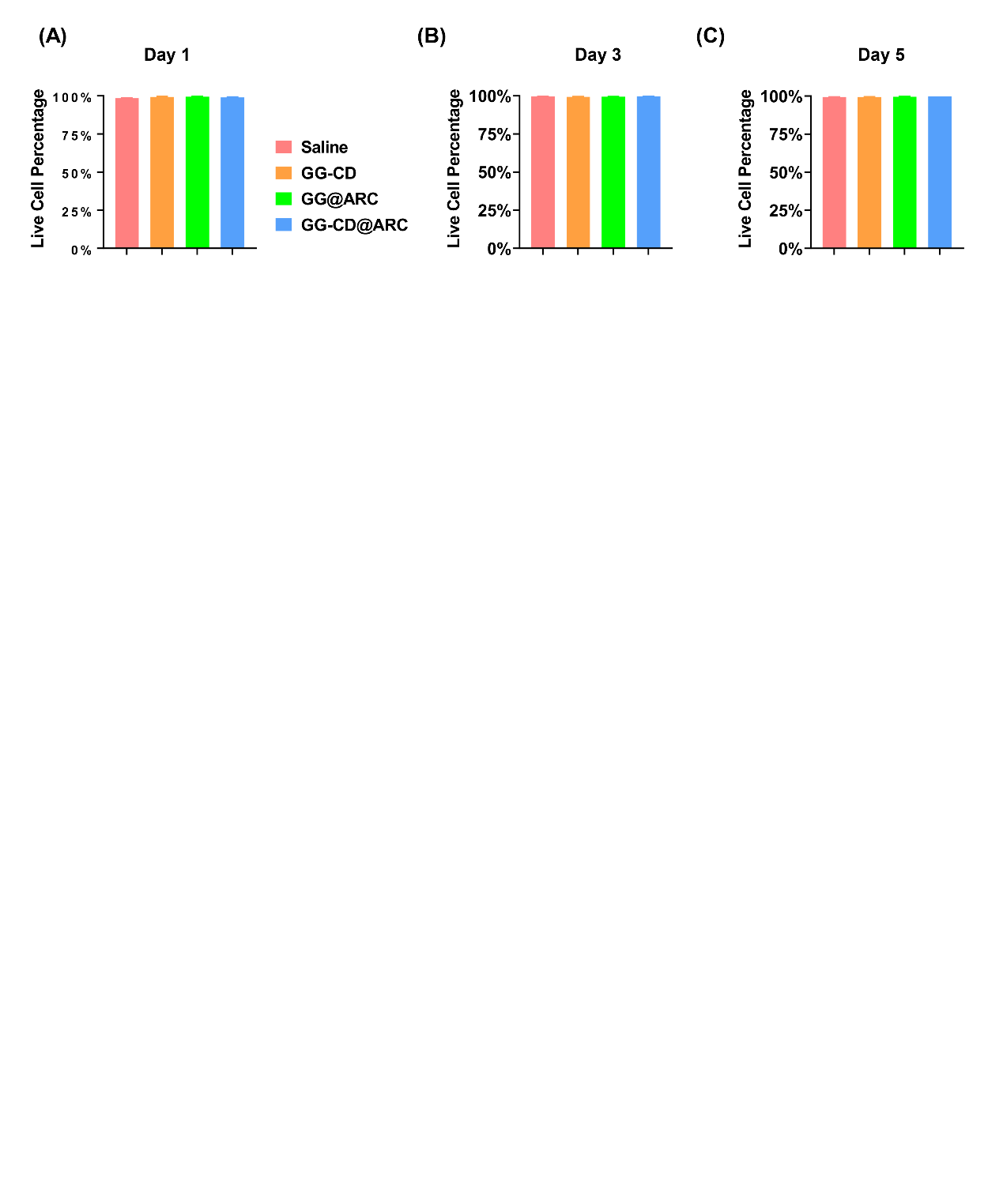


**Supplementary Figure 4.** Biocompatibility of GG-CD@ARC biological glue. (A-C) Quantification of the viability of cells cultured with leachate and stained with Live/Dead assays at day 1, 3, or 5 (n = 3). Values represent mean ± SD. Statistically, significant differences are indicated by ^#^ where *p* < 0.05, ^##^ where *p* < 0.01 compared with the CTRL group or * where *p* < 0.05, ** where *p* < 0.01 between the indicated groups.
