# Supplementary material for: Arctiin-reinforced antioxidant microcarrier antagonizes osteoarthritis progression
Source: J Nanobiotechnology. 2022 Jun 27;20:303. doi: 10.1186/s12951-022-01505-7 (PMC9235181; doi:10.1186/s12951-022-01505-7)
Supplement: Supplementary file 2 — Additional file 2. Table S1. Primers used for real-time PCR. [file 12951_2022_1505_MOESM2_ESM.docx]

**Supplementary Table 1.** Primers used for real-time PCR

| **Gene** | **Forward Primer sequence (5’-3’)** | **Reverse Primer sequence (5’-3’)** |
| --- | --- | --- |
| *COL2A1* | TGGACGCCATGAAGGTTTTCT | TGGGAGCCAGATTGTCATCTC |
| *ACAN* | ACTCTGGGTTTTCGTGACTCT | ACACTCAGCGAGTTGTCATGG |
| *MMP13* | ACTGAGAGGCTCCGAGAAATG | GAACCCCGCATCTTGGCTT |
| *ADAMTS5*  *SOD1*  *SOD2*  *CAT*  *GPX1* | ACTACGATGCAGCTATCCTGT  GGTGGGCCAAAGGATGAAGAG  GGGGATTGATGTGTGGGAGCACG  TGGGATCTCGTTGGAAATAACAC  TATCGAGAATGTGGCGTCCC | GTCCCAACGTCTGCCATTC  CCACAAGCCAAACGACTTCC  AGACAGGACGTTATCTTGCTGGGA  TCAGGACGTAGGCTCCAGAAG  TCTTGGCGTTCTCCTGATGC |
| *NRF2* | TCAGCGACGGAAAGAGTATGA | CCACTGGTTTCTGACTGGATGT |
| *GAPDH* | AGAAAAACCTGCCAAATATGATGAC | TGGGTGTCGCTGTTGAAGTC |
